# Host‐induced gene silencing of a regulator of G protein signalling gene (*VdRGS1*) confers resistance to *Verticillium* wilt in cotton

**DOI:** 10.1111/pbi.12900

**Published:** 2018-03-05

**Authors:** Jun Xu, Xinyu Wang, Yongqing Li, Jianguo Zeng, Guilin Wang, Chaoyang Deng, Wangzhen Guo

**Affiliations:** ^1^ State Key Laboratory of Crop Genetics & Germplasm Enhancement Nanjing Agricultural University Nanjing Jiangsu China; ^2^ College of Life Sciences Nanjing Agricultural University Nanjing Jiangsu China

**Keywords:** regulator of G protein signalling, pathogenicity, host‐induced gene silencing, *Verticillium dahliae*, cotton (*Gossypium hirsutum*)

## Abstract

*Verticillium* wilt (VW), caused by soil‐borne fungi of the genus *Verticillium*, is a serious disease affecting a wide range of plants and leading to a constant and major challenge to agriculture worldwide. Cotton (*Gossypium hirsutum*) is the world's most important natural textile fibre and oil crop. VW of cotton is a highly devastating vascular disease; however, few resistant germplasms have been reported in cotton. An increasing number of studies have shown that RNA interference (RNAi)‐based host‐induced gene silencing (HIGS) is an effective strategy for improving plant resistance to pathogens by silencing genes essential for the pathogenicity of these pathogens. Here, we have identified and characterized multifunctional regulators of G protein signalling (RGS) in the *Verticillium dahliae* virulence strain, Vd8. Of eight *VdRGS* genes, *VdRGS1* showed the most significant increase in expression in *V. dahliae* after treating with the roots of cotton seedlings. Based on the phenotype detection of *VdRGS1* deletion and complementation mutants, we found that *VdRGS1* played crucial roles in spore production, hyphal development, microsclerotia formation and pathogenicity. Tobacco rattle virus‐mediated HIGS in cotton plants silenced *VdRGS1* transcripts in invaded *V. dahliae* strains and enhanced broad‐spectrum resistance to cotton VW. Our data demonstrate that *VdRGS1* is a conserved and essential gene for *V. dahliae* virulence. HIGS of *VdRGS1* provides effective control against *V. dahliae* infection and could obtain the durable disease resistance in cotton and in other VW‐susceptible host crops by developing the stable transformants.

## Introduction

*Verticillium* wilt (VW), caused by a soil‐borne hemibiotrophic fungus, *Verticillium dahliae* Klep, leads to vascular wilt disease and poses a destructive threat to more than 200 plant species (Bolek *et al*., 2005; Fradin and Thomma, 2006; Koroleva *et al*., 2001). This pathogen is particularly difficult to control, because it not only has a highly aggressive pathogenicity, but also persists in the host xylem vessels or soil as resting structures, called microsclerotia, for several years (Pegg and Brady, 2002; Klosterman *et al*., 2009; Luo *et al*., 2014; Wang *et al*., 2016). When the roots of host plants are challenged with *V. dahliae*, the microsclerotia germinate, and then produce conidia, mycelia and melanized dormancy microsclerotia, resulting in the blockage of plant vessels and VW (Fitzell *et al*., 1980; Gerik and Huisman, 1988). In addition, the broad range of *V. dahliae* hosts makes crop rotation ineffective.

The most sustainable way to control VW disease is the use of resistant cultivars. However, available natural resistance resources for VW are very limited, meaning that the breeding of resistant cultivars via conventional approaches is a challenge. To alleviate the negative impact of wilt disease, a continuous search for alternative control strategies is required. In the past 10 years, heterologous expression of foreign genes and the silencing of endogenous genes known as ‘susceptible genes’ have been used to improve crop resistance (Gao *et al*., 2016; Song *et al*., 2017). In recent years, an alternative technique called host‐induced gene silencing (HIGS), or host‐delivered RNAi, has been shown to successfully lead to the expression of antisense or hairpin RNAi constructs, or other forms of short interfering RNA molecules, to directly silence a gene of interest and, therefore, engineer plants that are resistant to plant viruses, insects and bacterial and fungal pathogens (Baum *et al*., 2007; Cheng *et al*., 2015; Waterhouse and Fusaro, 2006; Zhang *et al*., 2016). Several successful examples of improved plant resistance by the silencing of genes that are essential for pathogens have been reported. For example, fungal cytochrome P450 lanosterol C‐14α‐demethylase (*CYP51*) genes are vital for ergosterol biosynthesis and infection, and transgenic *Arabidopsis* and barley expressing a double‐stranded RNA targeting all three fungal *CYP51* genes exhibited complete immunity to *Fusarium* species (Koch *et al*., 2013). Chitin synthase *Chs3b* is an essential virulence gene in *Fusarium graminearum* (Fg), and HIGS of *Chs3b* confers durable resistance to *Fusarium* head blight and seedling blight in wheat (Cheng *et al*., 2015). *PsCPK1* is a protein kinase A (PKA) catalytic subunit gene and is highly expressed at the early infection stage of *Puccinia striiformis f. sp. tritici* (Pst), and the barley stripe mosaic virus (BSMV)‐mediated HIGS of *PsCPK1* significantly impaired the length of infection hyphae and disease phenotypes. In addition, two transgenic lines expressing RNAi targeting *PsCPK1* enhanced the resistance of wheat to stripe rust (Qi *et al*., 2017). Also, HIGS of fungal genes has been shown to control the growth and virulence of *V. dahliae* and suppress disease in tomato, *Arabidopsis* and cotton (Song and Thomma, 2016; Zhang *et al*., 2016). *VdH1* is associated with melanized microsclerotia production and pathogenicity in the *V. dahliae*, and HIGS of this gene confers effective resistance to the cotton wilt disease (Zhang *et al*., 2016). Furthermore, transient assays of tobacco rattle virus (TRV)‐based virus‐induced gene silencing (VIGS) have successfully been used to silence the *Ave1* gene in tomato and *Arabidopsis* and led to a clear inhibition of VW disease (Song and Thomma, 2016).

G protein (heterotrimeric guanine nucleotide binding protein) signalling, which functions by sensing extracellular signals and integrating them into intrinsic signal transduction pathways, is one of the most important signalling mechanisms in fungi (Malbon, 2005). Regulators of G protein signalling (RGS), a family of multifunctional and diverse signalling proteins, contain a conserved domain of 120 amino acids and function as negative regulators of G protein‐coupled signalling pathways (Cavalli *et al*., 2000; Dohlman *et al*., 1996; Siderovski *et al*., 1996). RGS proteins are an indispensable component of the G protein‐coupled receptor–effector complex. They commonly serve as GTPase accelerators that promote GTP hydrolysis through Gα subunits, thereby rapidly switching off G protein‐coupled signalling pathways and interdicting the downstream signals (De Vries *et al*., 2000; Siderovski and Willard, 2005). The first RGS protein to be discovered, Sst2, was confirmed as a negative regulator of pheromone signalling in the yeast, *Saccharomyces cerevisiae* (Dohlman *et al*., 1996). Subsequently, the functions of RGS proteins have been elucidated in a variety of fungi, such as the plant pathogenic fungi *Magnaporthe oryzae* (Liu *et al*., 2007; Zhang *et al*., 2011), *Fusarium verticillioides* (Mukherjee *et al*., 2011), and *Gibberella zeae* (Park *et al*., 2012), the model saprophytic fungus, *Aspergillus nidulans* (Yu, 2006), and the insect pathogenic fungus, *Metarhizium anisopliae* (Fang *et al*., 2007). RGS proteins are involved in a complex process to control appressorium differentiation and penetration, asexual/sexual development, and pathogenicity (Zhang *et al*., 2011).

Cotton (*Gossypium hirsutum*) is the world's most important natural textile fibre and oil crop. Cotton VW is one of the most serious vascular diseases and leads to severe reductions in cotton mass and yield worldwide (Bolek *et al*., 2005; Cai *et al*., 2009). Resistance resources for VW in cotton are very limited, and VW has not been effectively controlled by the breeding of resistant varieties via conventional approaches. Several virulence factors of *V. dahliae* have been identified for use in the improvement of disease resistance in cotton (Zhang *et al*., 2016). Nevertheless, the functions and molecular mechanisms of RGS and RGS‐like proteins in *V. dahliae* and their potential application in improving cotton disease resistance remain largely unknown. In this study, we first systematically identified and surveyed the RGS and RGS‐like genes in the *V. dahliae* virulence strain, Vd8. We found that *VdRGS1* had the most significant expression increase in *V. dahliae* after the roots of cotton seedlings were induced, when compared to other *VdRGS* genes. Targeted gene deletion of *VdRGS1* in *V. dahliae* strain Vd8 led to a drastic reduction in fungal spore production, aerial hyphae development, microsclerotia formation and pathogenicity. Further, TRV‐based silencing in cotton compromised *V. dahliae VdRGS1* expression and significantly enhanced VW resistance. This study provides a reference for the development of stable transgenic plants using a HIGS‐mediated *VdRGS1* strategy to improve resistance to *V. dahliae* in cotton and other VW‐susceptible host crops.

## Results

### Identification and expression patterns of the RGS genes in *V. dahliae*

**Figure 1:**
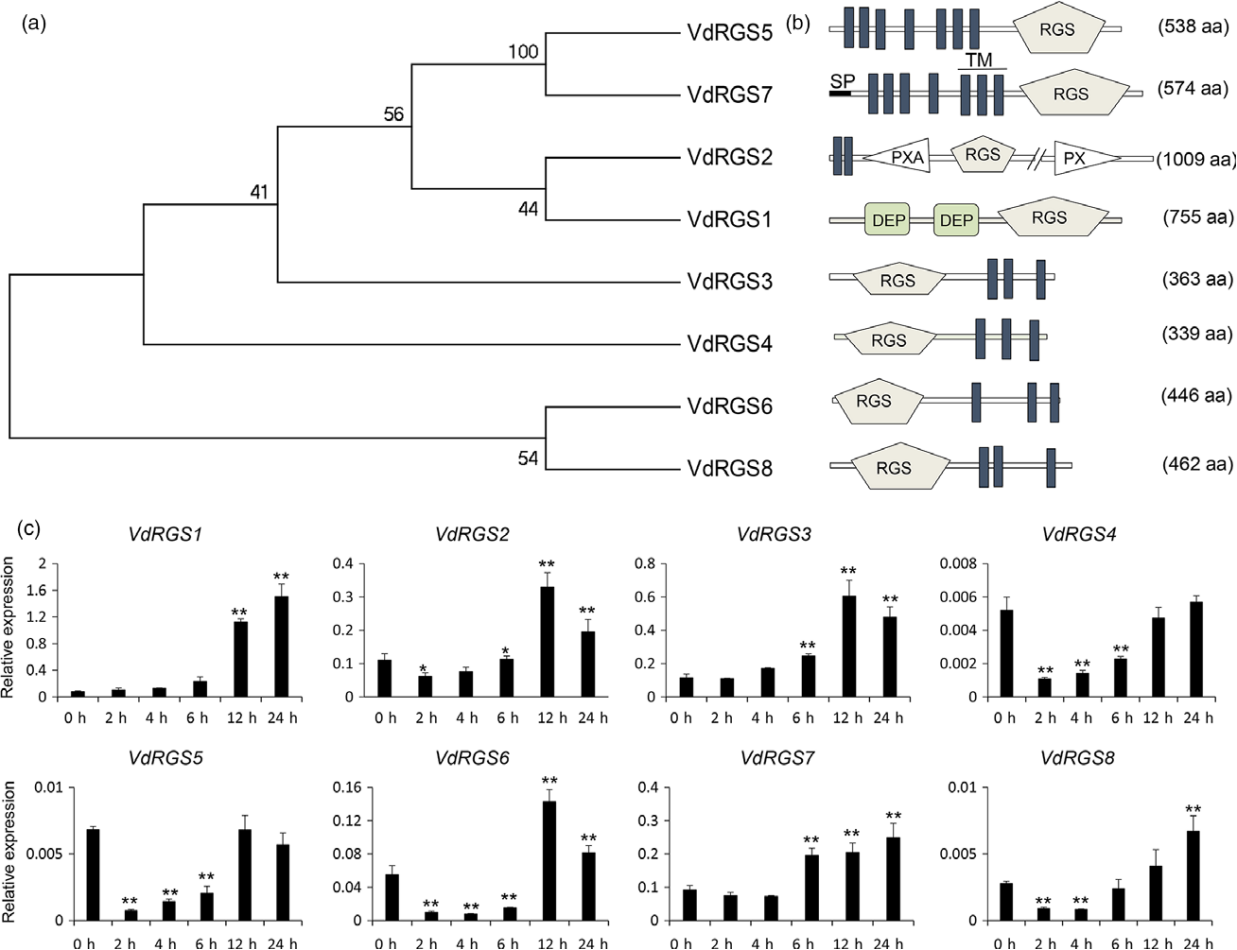
Identification and expression of RGS genes in *Verticillium dahlia*. (a) Phylogenetic relationship of the eight RGS genes in *V. dahliae*. (b) Prediction of the domains of the eight RGS proteins. The eight RGS proteins not only have the RGS domain but also contain other functional domains. (c) Expression of eight RGS genes in *V. dahliae* induced by the roots of cotton seedlings at different time points. The roots of 2‐week‐old cotton seedlings are used to induce the *V. dahliae. Verticillium dahliae* spore suspensions induced for 0, 2, 4, 6, 12 and 24 hours (h) were collected separately and used for RNA isolation. RGS gene expressions were examined by qRT–PCR. The data represent the mean ± SD of three samples from three independent tests at each time point. ‘*’: significant difference at *P *< 0.05; ‘**’: significant difference at *P* < 0.01.

RGS proteins were involved in a complex process to control the fungal development and pathogenicity. Based on the RGS (PF00615) domain and the whole‐genome sequence of *V. dahliae* VdLs17 (http://genome.jgi.doe.gov/Verda1/Verda1.home.html), eight RGS genes were identified and the sequences were further confirmed by cloning them from cotton *V. dahliae* virulence strain Vd8. The eight genes were named individually as *VdRGS1* (ortholog of VDAG_00683 in VdLs17), *VdRGS2* (VDAG_10098), *VdRGS3* (VDAG_00979), *VdRGS4* (VDAG_02225), *VdRGS5* (VDAG_09977), *VdRGS6* (VDAG_02523), *VdRGS7* (VDAG_08194), and *VdRGS8* (VDAG_07045) (GenBank accession numbers: MG583845‐MG583852). With the exception of the common RGS domain, the eight VdRGSs showed a high amino acid diversity (Figure 1a). Only VdRGS1 possessed tandem Dishevelled, EGL‐10 and Pleckstrin (DEP) domains (Figure 1b), which mainly function in the spatial and temporal control of diverse signal transduction events by recruiting proteins to the plasma membrane and regulating subcellular targeting. The DEP domains may also interact with various partners located at the membrane, including phospholipids and membrane receptors, and their bindings are subject to regulation (Consonni *et al*., 2014; Ramanujam *et al*., 2012).

To further ascertain the functions of the RGS genes in *V. dahliae*, we analysed the expression patterns of the eight RGS genes by collecting *V. dahliae* spore suspensions induced with the roots of cotton seedlings at different time points. As shown in Figure 1c, six RGS genes showed increased expression levels; only *VdRGS4* and *VdRGS5* did not. Of the six, *VdRGS1* showed the most significant increase in expression, with a 14‐fold increase at 12 hours (h) and a 20‐fold increase at 24 h, compared to its expression in *V. dahliae* untreated with the roots of cotton seedlings. Other five *VdRGS*s also showed significant increases in expression: *VdRGS2* was threefold higher at 12 h, *VdRGS3* was fivefold higher at 12 h, *VdRGS6* was 2.6‐fold higher at 12 h, *VdRGS7* was 2.7‐fold higher at 24 h, and *VdRGS8* was 2.4‐fold higher at 24 h. These results indicate that *VdRGS1* plays an important role in the virulence of *V. dahliae*.

### *VdRGS1* plays crucial roles in spore germination, hyphal development, spore production and microsclerotia formation

**Figure 2:**
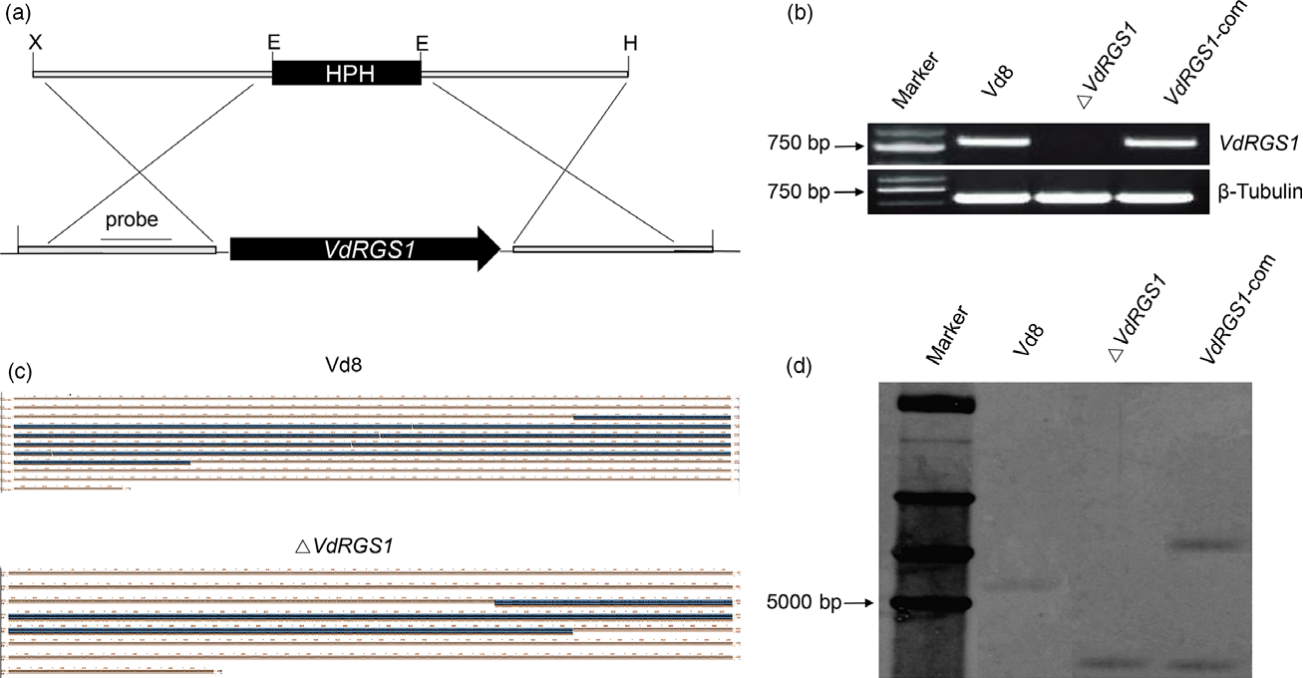
Strategies and confirmation of *VdRGS1* deletion and complementation. (a) Schematic diagram of *VdRGS1* replacement by HPH via homologous recombination. (b) Confirmation of *VdRGS1* deletion and complementation mutants by RT‐PCR using *VdRGS1* gene‐specific primers. The β‐tubulin gene was used as the internal control. (c) Confirmation of *VdRGS1* deletion by DNA sequencing of the *VdRGS1* locus. (d) Confirmation of *VdRGS1* deletion by Southern blot analysis. Total genomic DNA wild‐type strain Vd8 (WT), Δ*VdRGS1* and VdRGS1‐com were digested with *Sac* I and subjected to Southern blot analysis. The probe location is indicated in Figure 2a.

To better ascertain the functions of *VdRGS1* in *V. dahliae*,* VdRGS1* deletion mutants (named Δ*VdRGS1*) replaced by a selective marker gene (the bacterial phosphotransferase gene, HPH) were generated via homologous recombination method (Figure 2a), and the resulting Δ*VdRGS1* strain was further confirmed by RT‐PCR (Figure 2b), DNA sequencing of the *VdRGS1* gene locus (Figure 2c) and Southern hybridization (Figure 2d). Thereafter, the complemented strain *VdRGS1*‐com was generated by reintroducing the *VdRGS1* gene containing the native promoter and terminator sequences into Δ*VdRGS1* mutants (Figure 2b,d).

**Figure 3:**
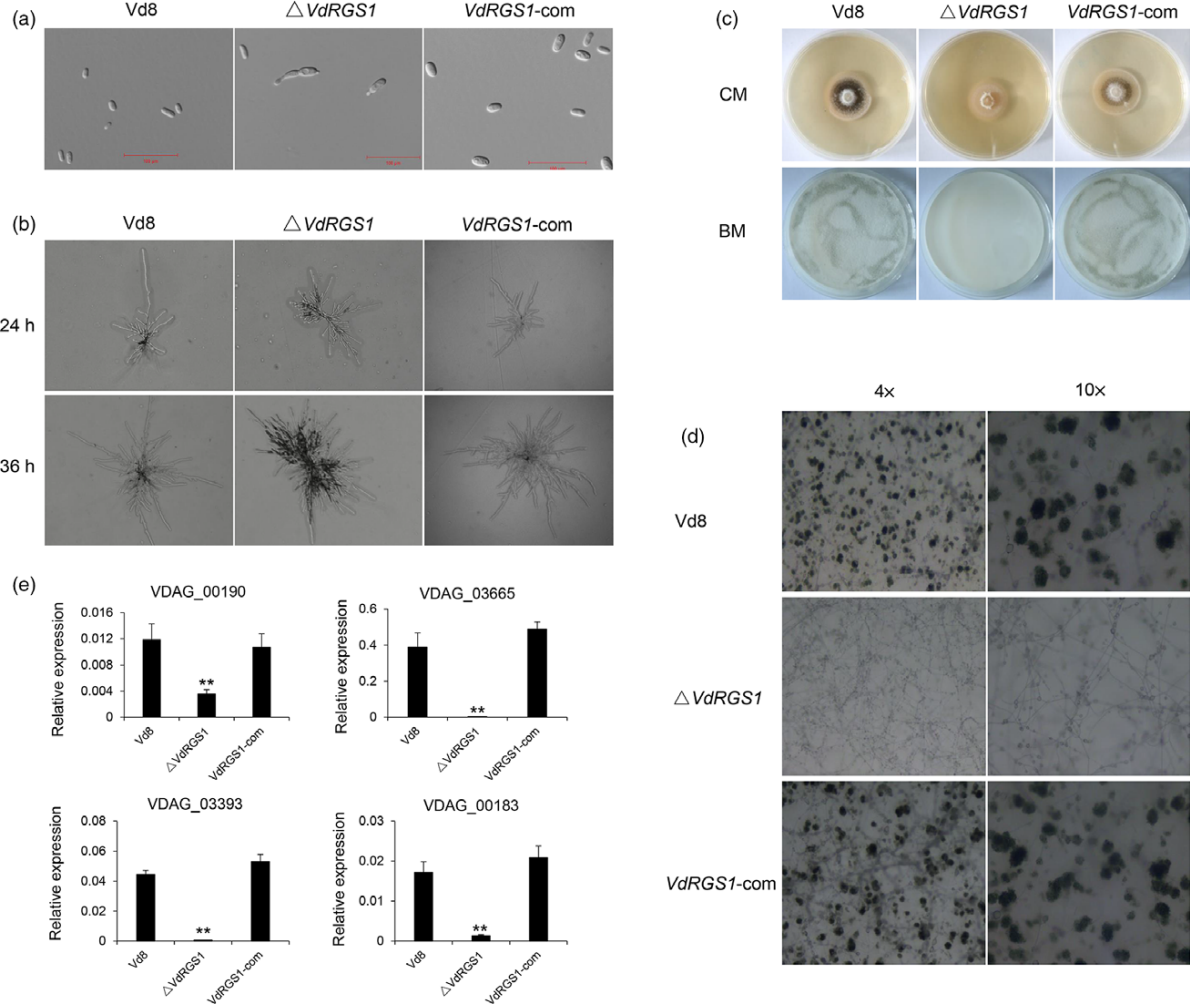
Effects of *VdRGS1* deletion on spore germination, hyphal development, spore production and microsclerotia formation. (a) Microscopy of spores of Δ*VdRGS1* and *VdRGS1*‐com compared to Vd8. Bars = 100 μm. (b) Microscopy of hyphal growth from single spore of Vd8, Δ*VdRGS1* and *VdRGS1*‐com strains cultured on CM agar plates. Photographs were taken at 24 and 36 h postincubation, respectively. (c) The strains of Vd8, Δ*VdRGS1* and *VdRGS1*‐com from single spore cultured in the CM medium for 5 days at 24 °C. Further, observation of microsclerotia produced in these three strains on BM medium. The spore suspensions of the Vd8, Δ*VdRGS* 1 and *VdRGS* 1‐com were diluted to 1 × 10^5^ spores/mL, and sprayed on cellulose membrane placed onto BM plates and incubated at 24 °C for 5 days. (d) Microscopy of microsclerotia produced by Vd8, Δ*VdRGS1* and *VdRGS1*‐com 5 days postinoculation under 4× and 10× mean objective magnification of microscope. (e) Expression of genes involved in melanin biosynthesis in each of Vd8, Δ*VdRGS1* and *VdRGS1*‐com strains. The data represent the mean ± SD of three samples from three independent tests. ‘**’: significant difference at *P* < 0.01.

To analyse the effects of *VdRGS1* on fungal growth and spore germination, the Δ*VdRGS1*,* VdRGS1*‐com and wild‐type strain Vd8 were incubated on complete medium (CM), and an equal number of 1000 spores of different strains were observed under a microscope, respectively. The spores of Vd8 and *VdRGS1*‐com strains showed normal development, yet more than a third of spores in the Δ*VdRGS1* strain were germination in advance at the early stage (Figures 3a and S1a). Further, the hyphae of the Vd8 and *VdRGS1*‐com strains displayed radial and fast growth and formed flat mycelia on agar plates, yet the Δ*VdRGS1* strain developed dumbbell‐shaped hyphae (Figure 3b). In addition, *VdRGS1*‐com and Vd8 shared identical phenotypes in microsclerotia formation both on CM and on potato dextrose agar (PDA) media, while Δ*VdRGS1* exhibited a light colour, indicating that microsclerotia production was significantly affected in this mutant (Figure S2a). Moreover, *VdRGS1* also significantly affected conidiospore production in *V. dahliae*. We detected 1000 spores of each of the three different strains incubated in Czapek–Dox medium (CPK) for 7 days and found that the spore concentration of the Δ*VdRGS1* mutant (7.23 ± 0.64 × 10^6^) was significantly less than that of the wild‐type Vd8 (20.3 ± 0.61 × 10^6^) and the *VdRGS1*‐com (18 ± 1 × 10^6^) strains (Figures S2b and 2c).

To better confirm the function of *VdRGS1* in the microsclerotia, we investigated microsclerotia formation in the three different strains on CM and basal medium (BM). After growing on CM medium for 5 days, the wild‐type Vd8 and the *VdRGS1*‐com strains produced obvious melanized microsclerotia, yet microsclerotia production in the Δ*VdRGS1* strains was clearly inhibited (Figure 3c). Next, the production of microsclerotia was further examined by uniformly spraying a conidial suspension onto a cellulose membrane that was overlaid onto the BM medium. Very few pigmented colonies appeared 5 days postinoculation (dpi) in the Δ*VdRGS1* mutant, relative to those observed the wild‐type Vd8 and *VdRGS1*‐com strains (Figure 3c). Microscopic analyses revealed that the *VdRGS1*‐com and Vd8 strains exhibited similar levels of melanin, while there were smaller microsclerotial masses produced in the Δ*VdRGS1* strain (Figure 3d). To further determine whether *VdRGS1* regulates the expression of genes involved in melanin biosynthesis, several key genes related to melanin biosynthesis in *V. dahliae* (Wang *et al*., 2016; Xiong *et al*., 2015), including polyketide synthase (VDAG_00190), tetrahydroxynaphthalene reductase (VDAG_03665), scytalone dehydratase (VDAG_03393), versicolorin reductase (VDAG_00183) and acetyl‐CoA carboxylase (VDAG_03674), were selected for qRT‐PCR analyses. Expression data showed that with the exception of VDAG_03674, other four genes were obviously down‐regulated in the Δ*VdRGS1* mutant compared with the Vd8 and *VdRGS1*‐com strains (Figure 3e). Especially, the transcript levels of VDAG_03393 and VDAG_03665 were down‐regulated 57‐ and 104‐fold, respectively, in the Δ*VdRGS1* mutant. Taken together, *VdRGS1* plays crucial roles in spore production and microsclerotia formation of *V. dahliae*.

### *VdRGS1* is required for full virulence in *V. dahliae*

**Figure 4:**
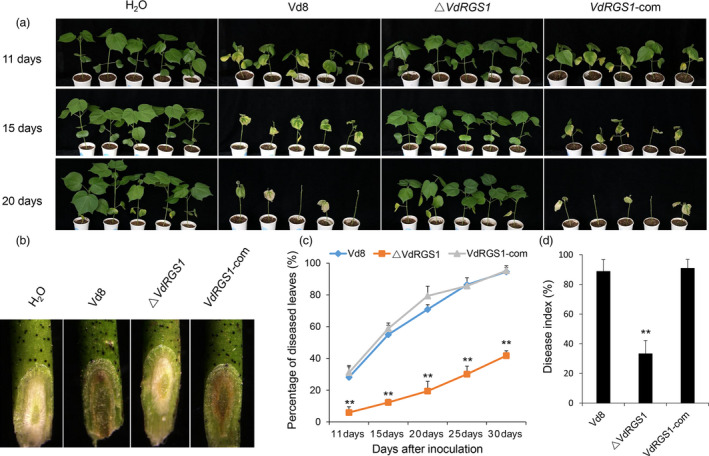
Virulence tests of Δ*VdRGS* 1. (a) Disease symptoms of cotton plants (Junmian 1) infected with Δ*VdRGS* 1 compared with wild‐type Vd8 and *VdRGS1*‐com strains. Photographs were taken at 11, 15 and 20 days postinoculation. (b) Vascular discoloration of cotton plants inoculated with indicated strains. Plants were inoculated with *Verticillium dahliae* spore suspensions (10^7^ conidia/mL) for 15 days, and then, the stems were cut and photographed by stereoscope (Olympus MVX10, Tokyo, Japan). (c) The percentage of diseased leaves inoculated with Δ*VdRGS1* compared with wild‐type Vd8 and *VdRGS1*‐com strains. (d) Disease index (DI) of cotton plants inoculated the wild‐type Vd8, Δ*VdRGS1* and *VdRGS1*‐com at 20 dpi. These experiments were repeated for three times using at least 40 seedlings per treatment. Error bars show the standard deviation of three biological replicates. Asterisks indicate statistically significant differences in the percentage of diseased leaves and the DI of plants treated with Δ*VdRGS1* and *VdRGS1*‐com mutants and the wild‐type Vd8 strain, as determined by Student's *t*‐tests (*P < 0.05, **P < 0.01).

To investigate the role of *VdRGS1* in pathogenicity, seedlings of *G. hirsutum* cv. Junmian 1, an accession susceptible to *V. dahliae*, were inoculated with the spore suspension of the Vd8, Δ*VdRGS1* and *VdRGS1*‐com strains. About 10 days after inoculation, the seedlings of the Junmian 1 plants challenged with Vd8 and *VdRGS1*‐com strains showed similar disease symptoms, yet the plants inoculated with the Δ*VdRGS1* mutants had no distinct phenotypic difference with the H_2_O‐treated control plants. After a further 10 days, almost all the true leaves were defoliated in the seedlings inoculated with Vd8 and *VdRGS1*‐com strains, while the VW disease symptoms exhibited very few in the cotyledons of plants inoculated with the Δ*VdRGS1* mutants (Figure 4a).

To further examine the diminished pathogenicity of the Δ*VdRGS1* mutant, the stem vascular bundles of plants inoculated with Vd8, Δ*VdRGS1* and *VdRGS1*‐com strains were assessed using a stereoscope by sampling the seedlings at 15 dpi. The results indicated that the stem vascular bundles of plants inoculated with Vd8 and *VdRGS1*‐com strains exhibited melanin and microsclerotia accumulation, yet the colour of vascular bundles in the plants infected with the Δ*VdRGS1* mutant was similar to H_2_O‐treated control plants (Figure 4b). In addition, we used at least 40 plants per treatment to calculate the rate of diseased leaves and the disease index (DI). As a result, the rate of average diseased leaves to total leaves was approximately 40% after 30 days inoculation with Δ*VdRGS1* mutants, while nearly 100% plants inoculated with Vd8 and *VdRGS1*‐com strains showed leaves wilting or defoliation (Figure 4c). Also, the DI of cotton plants challenged with different strains at 20 dpi was consistent with the data of rate of average diseased leaves (Figure 4d). The disease resistance of the seedlings inoculated with Δ*VdRGS1* mutants was significantly enhanced compared to these inoculated with wild‐type strain Vd8 (*P* < 0.01). These observations indicate that *VdRGS1* is an essential gene in the pathogenicity of *V. dahliae* and its deletion significantly affects the fungus virulence.

### TRV‐based HIGS works well in the cotton plants

An increasing number of studies have shown that the virus‐mediated HIGS in planta can generate RNAi of the pathogen and control pathogen infection in a variety of plants (Panwar *et al*., 2013; Qi *et al*., 2017; Song and Thomma, 2016). To investigate whether TRV‐based HIGS can be utilized against *V. dahliae* in the cotton plants, with *GFP* as a marker gene, we constructed a GFP expression vector, which was transformed into spores of the wild‐type *V. dahliae* strains Vd8 and V991 using the ATMT method. Subsequently, we further screened the mutants and acquired stable V991‐GFP strains, which not only had obvious GFP signals, but also possessed highly aggressive pathogenicity (Figure S3). Next, the V991‐GFP strain was inoculated into cotton plants, which were treated with a TRV: *GFP* vector, constructed via cloning of a specific fragment of the GFP gene. The vector was designed to silence endogenous genes of the V991‐GFP strain, based on TRV‐based HIGS technology. The cotton seedlings without *Agrobacterium* infiltration (CK) and TRV: 00 were used as mock treatments and TRV: *GhCLA1* as a positive control to validate the efficiency of the VIGS assay.

**Figure 5:**
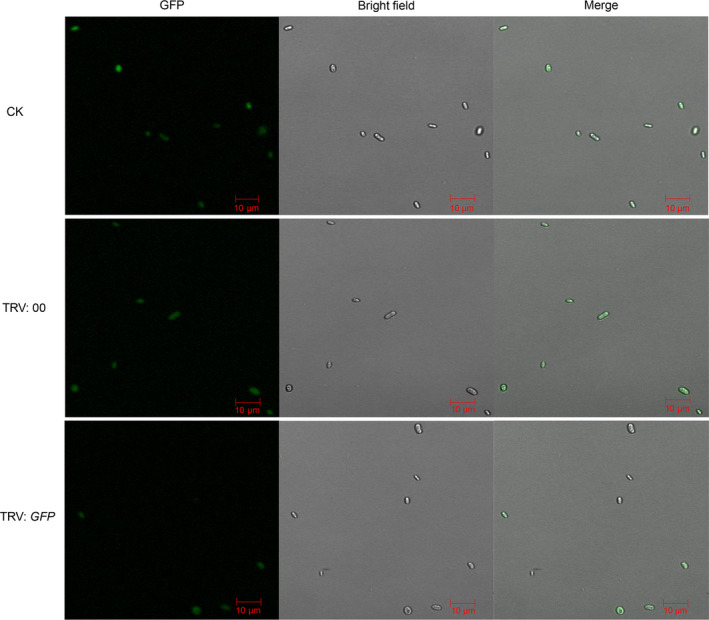
Effects of TRV‐based HIGS on GFP expression in spores of V991‐GFP strain. GFP expression in spores of V991‐GFP strain was observed after its challenge of cotton plant infiltrated with TRV: *GFP* construct. For spore recovery, stems of cotton plants infiltrated with TRV: 00, TRV: *GFP* constructs, respectively, were sampled at 15 days after V991‐GFP inoculation, then incubated on the CM medium at 24 °C for 3 days. The GFP fluorescence in recovered spores from different treatments was observed under the confocal microscopy. Bar = 10 μm.

As expected, the cotton leaves displayed an obvious photobleaching phenotype after 2 weeks agroinfiltration with the *GhCLA1* construct (Figure S4), suggesting that the VIGS technique worked well in our experimental operations. Subsequently, the CK, TRV: 00 and TRV: *GFP* plants were all challenged with the V991‐GFP strain via the dip‐infection method (Wang *et al*., 2011). At 15 dpi, the seedlings showed obvious leaf‐yellowing phenotypes, and the detected transcripts of *GFP* were significantly lower in the TRV: *GFP* plants (Figure S5). We individually observed the GFP fluorescence of 1000 spores from each of the treatments and found that all the spores in the CK and TRV: 00 plants had obvious GFP fluorescence, yet only about 50% spores in the TRV: *GFP* plants possessed detectable GFP signals (Figures 5 and S1b). Taken together, these results suggest that TRV‐based HIGS can work well to silence vital *V. dahliae* genes in the cotton plants.

### TRV‐based *VdRGS1* silencing in cotton inhibits VW disease

As described above, *VdRGS1* plays crucial roles in spore production, microsclerotia formation and pathogenicity in *V. dahliae*. Using a TRV‐based HIGS system, we investigated the functional role of this gene in cotton resistance to VW. Vd8 and V991 strains are two different physiological types of *V. dahliae*, but both possess highly aggressive pathogenicity to cotton plants. We cloned *VdRGS1* in the two strains and found the same gene sequence in each, indicating that the sequence of *VdRGS1* is conserved in different *V. dahliae* strains. We randomly selected four nonoverlapping fragments of *VdRGS1*, named *VdRGS1‐1*,* VdRGS1‐2*,* VdRGS1‐3* and *VdRGS1‐4*, to construct VIGS vectors for further TRV‐based HIGS analysis (Figure S6). When cotton plants infiltrated with TRV: *GhCLA1* displayed highly uniform bleaching in newly emerged leaves, the seedlings treated with all other vectors were inoculated with *V. dahliae* isolate Vd8 by dip infection. In parallel, the TRV‐based HIGS untreated plants were also challenged with the Δ*VdRGS1* mutants and H_2_O as controls.

**Figure 6:**
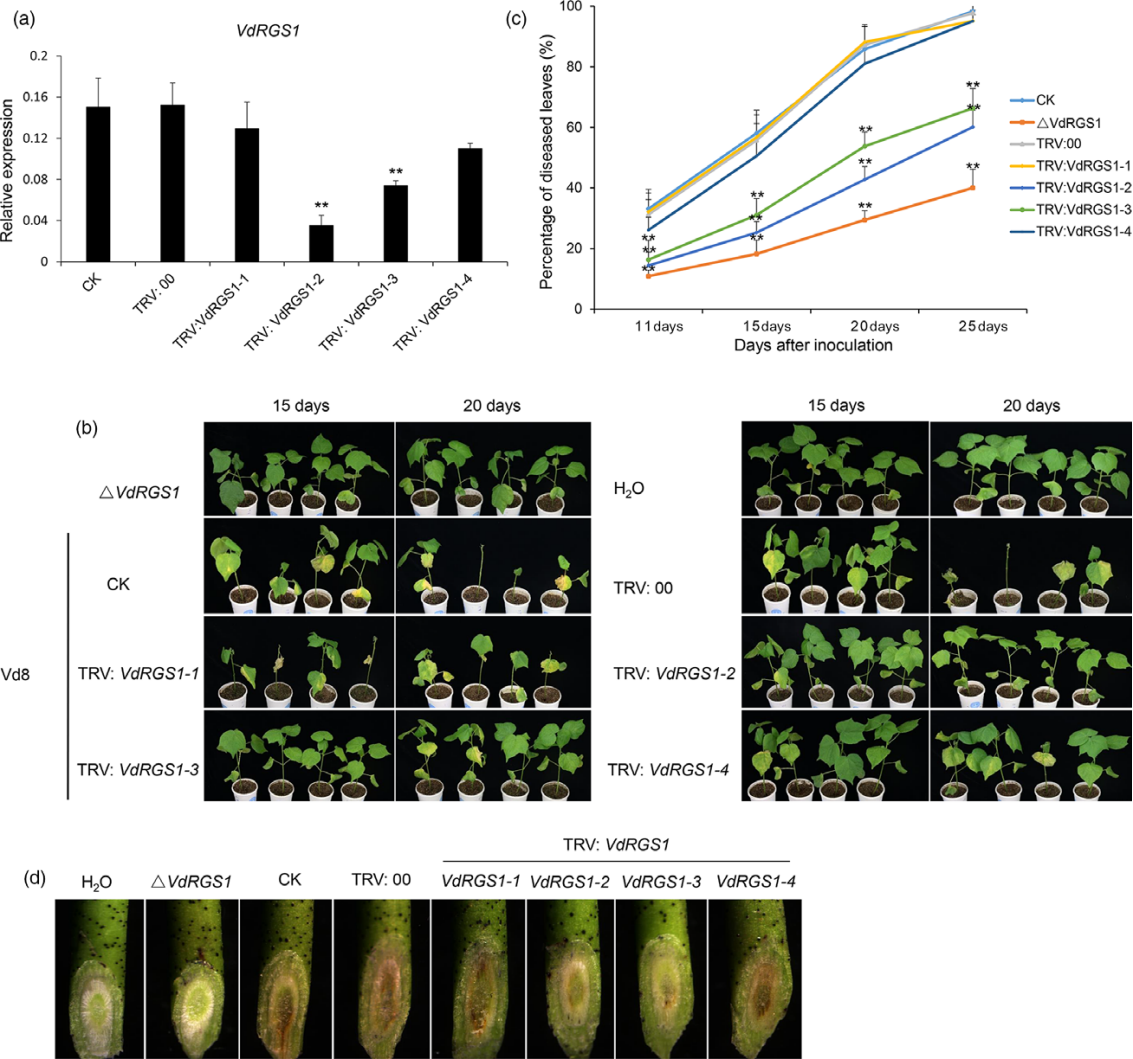
Effects of *VdRGS1* HIGS on cotton resistance to *Verticillium dahlia*e Vd8 infection. (a) Expression levels of *VdRGS1* in Vd8‐infected cotton plants infiltrated with various TRV:* VdRGS1* constructs. *VdRGS1‐1*,* VdRGS1‐2*,* VdRGS1‐3 and VdRGS1‐4* represent the different cDNA fragments within the *VdRGS1* coding region that were inserted in TRV vector. The data represent the mean ± SD of three samples from three independent tests. ‘*’: significant difference at *P* < 0.05; ‘**’: significant difference at *P* < 0.01. (b) Disease symptoms of the *VdRGS1* HIGS cotton plants infiltrated with TRV:* VdRGS1‐1*,* VdRGS1‐2*,* VdRGS1‐3*,* VdRGS1‐4* constructs compared with Δ*VdRGS* 1 infected and the controls. (c) The percentage of diseased leaves in TRV:* VdRGS1‐1*,* VdRGS1‐2*,* VdRGS1‐3*,* VdRGS1‐4* infiltrated cotton plants and the controls after *Vd8* inoculation. These experiments were repeated for three times using at least 40 seedlings per treatment. Error bars show the standard deviation of three biological replicates. Asterisks indicate statistically significant differences determined by Student's *t*‐tests (*P < 0.05, **P < 0.01). (d) Vascular discoloration in stems of different HIGS and control plants inoculated with V991‐GFP strain. Plants were inoculated with *V. dahliae* spore suspensions (10^7^ conidia/mL) and photographed by stereoscope (Olympus MVX10, Japan) at 15 dpi.

Ten days after inoculation, the seedlings of the control Junmian 1 plants showed obvious cotyledon wilting. After a further 5 days, plants began to exhibit obvious leaf‐yellowing phenotypes. We then randomly sampled stems above the cotyledons from plants with each of the VIGS treatments and these were incubated on PDA medium for RNA extraction and expression analysis of the target gene. Compared with controls, the expression levels of *VdRGS1* were significantly lower in the TRV: *VdRGS1‐2*‐ and TRV: *VdRGS1‐3*‐silenced plants; however, no distinct difference in expression was detected in *TRV: VdRGS1‐1*‐ and TRV: *VdRGS1‐4*‐silenced plants (Figure 6a). The inoculation test showed that the *TRV: VdRGS1‐1*‐ and TRV: *VdRGS1‐4*‐silenced plants displayed more severe wilting and yellowing symptoms, similar to the phenotype of the CK and TRV: 00 seedlings at 15 or 20 days after Vd8 inoculation. However, the TRV: *VdRGS1‐2*‐ and TRV: *VdRGS1‐3*‐silenced plants significantly enhanced the cotton VW resistance and showed fewer leaf‐yellowing phenotypes than the CK and TRV: 00 seedlings. In addition, there were very few disease symptoms detected in the cotyledons of plants treated with the Δ*VdRGS1* mutants, as with the plants treated with H_2_O (Figure 6b).

To better clarify the susceptibility of the control and VIGS plants to *V. dahliae* Vd8 and Δ*VdRGS1* strains, more than 40 plants per treatment were used to investigate the diseased and nondiseased leaves. The Junmian 1 seedlings without injection showed few wilted leaves and a ratio of diseased leaves to total leaves of approximately 35% after 25 days inoculation with the Δ*VdRGS1* mutants, yet more than 95% of the seedlings displayed leaf wilting or defoliation after 25 days inoculation with the Vd8 strain, indicating that Δ*VdRGS1* affected the pathogenicity of this strain. About 60% of the TRV: *VdRGS1‐2*‐ and TRV: *VdRGS1‐3*‐silenced plants showed wilting and yellowing symptoms; a value much lower than that of the control and *TRV: VdRGS1‐1*‐ and TRV: *VdRGS1‐4*‐silenced plants (Figure 6c). Also, the DI of the different VIGS plants after 20 days inoculation with Vd8 or the Δ*VdRGS1* strains was consistent with the data of rate of average diseased leaves (Figure S7a). In addition, the stem vascular bundles of the HIGS seedlings inoculated 15 days later with H_2_O, Vd8 and Δ*VdRGS1* strains were assessed using a stereoscope. As shown in Figure 6d, the stem vascular bundles of plants inoculated with Δ*VdRGS1* were similar to those of the H_2_O‐treated controls. The seedlings treated with Vd8, and the vascular bundles of the control, TRV:00 and TRV: *VdRGS1‐1*‐ and TRV: *VdRGS1‐4*‐silenced plants all exhibited melanin and microsclerotia accumulation, while the vascular bundles of the TRV: *VdRGS1‐2*‐ and TRV: *VdRGS1‐3*‐silenced plants only had a small distribution of melanin. Moreover, compromised immunity was also confirmed by fungal biomass quantification in stem sections of the inoculated plants. The fungal biomass quantifications revealed that less fungal biomass accumulated in plants inoculated with Δ*VdRGS1* mutants, and in the TRV: *VdRGS1‐2*‐ and TRV: *VdRGS1‐3* treated plants than in the TRV: 00 and TRV: *VdRGS1‐1*‐ and TRV: *VdRGS1‐4*‐silenced plants followed by inoculation with the wild‐type *V. dahliae* Vd8 (Figure S8a). These results suggest that HIGS of *VdRGS1* significantly inhibits Vd8 strain pathogenicity and enhances disease resistance in cotton.

**Figure 7:**
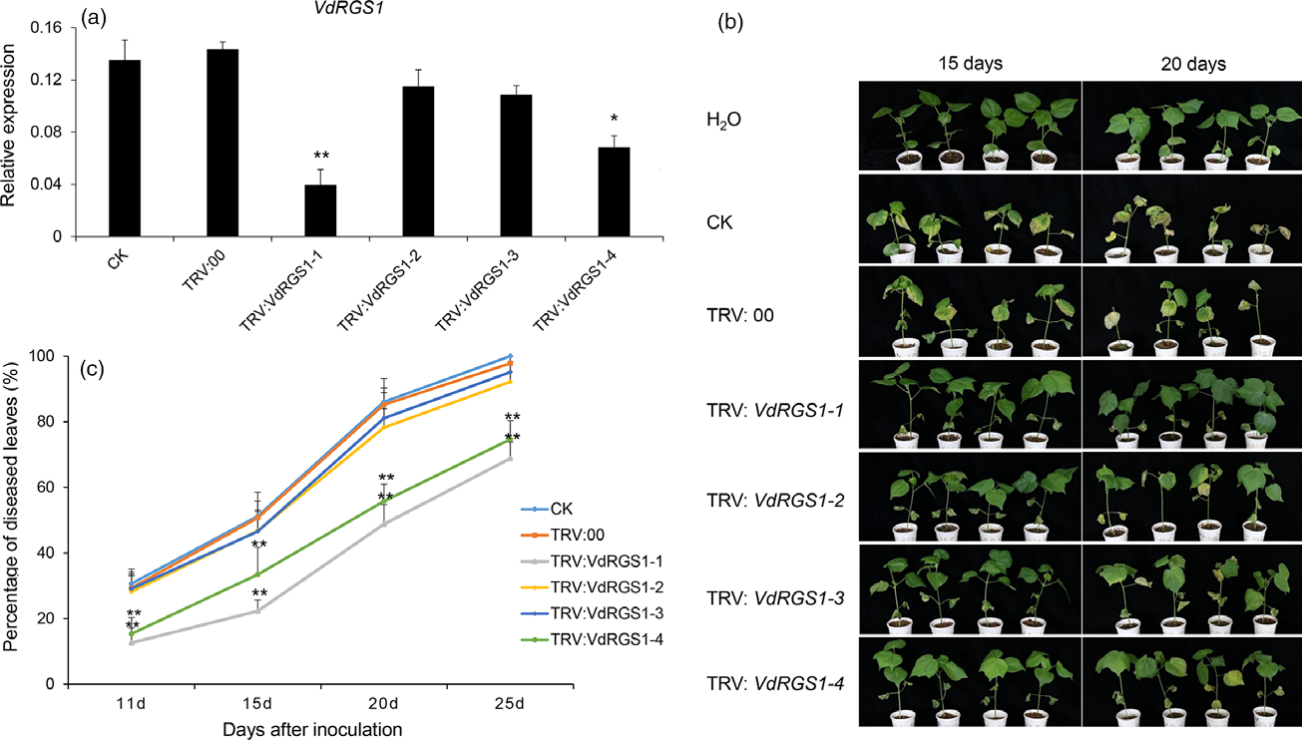
Effects of *VdRGS1* HIGS on cotton resistance to *Verticillium dahlia*e V991 infection. (a) Expression levels of the *VdRGS1* in the invaded V991 of different VIGS plants and compared with the TRV: 00 seedlings. Fifteen days after V991 inoculation, the stems of the different treatments were sampled and incubated on the CM medium at 24 °C for 3 days, and the strains were used for RNA extraction and qRT‐PCR analysis. The data represent the mean ± SD of three samples from three independent tests at each time point. ‘*’: significant difference at *P* < 0.05; ‘**’: significant difference at *P* < 0.01. (b) Disease symptoms of the *VdRGS1‐1*,* VdRGS1‐2*,* VdRGS1‐3*,* VdRGS1‐4*‐silenced, TRV: 00 and CK plants at 20 and 25 days after *V. dahliae* V991 inoculation. (c) The percentage of diseased leaves of the *VdRGS1‐1*,* VdRGS1‐2*,* VdRGS1‐3*,* VdRGS1‐4*‐silenced plants and controls after V991 inoculation. These experiments were repeated using at least 40 seedlings per treatment. Error bars show the standard deviation of three biological replicates. Asterisks indicate statistically significant differences in the percentage of diseased leaves between treated plants and TRV: 00 controls, as determined by Student's *t*‐tests (*P < 0.05, **P < 0.01).

To further validate whether TRV‐based HIGS can enhance resistance to VW caused by different physiological types of the *V. dahliae*, Junmian 1 seedlings infiltrated with different constructs via *Agrobacterium* were challenged with *V. dahliae* strain V991. In parallel, we also performed qRT‐PCR to analyse the expression of *VdRGS1* in the different VIGS plants and found that the transcripts of *VdRGS1* were significantly lower in the *TRV: VdRGS1‐1*‐ and TRV: *VdRGS1‐4*‐silenced plants (Figure 7a), and their resistance to cotton VW significantly enhanced (Figure 7b). Furthermore, the percentage of diseased leaves in *TRV: VdRGS1‐1*‐ and TRV: *VdRGS1‐4*‐silenced plants was significantly lower than that in controls and reached approximately 65% at 25 dpi, yet in controls and other VIGS plants with low silencing efficiency, almost 95% of leaves displayed wilting or defoliation (Figure 7c). Similarly, the DI of the different VIGS plants after 20 days inoculation with V991 strains was in agreement with the data of rate of average diseased leaves (Figure S7b). These data are further supported by fungal biomass quantification in stem sections of the inoculated plants (Figure S8b). Taken together, HIGS of *VdRGS1* provides effective control against *V. dahliae* infection. High silencing efficiency of *VdRGS1* with stable transformation could enhance broad‐spectrum resistance to cotton VW.

## Discussion

### Characterization of RGS in fungi

The G proteins are crucial components that sense and relay external cues into the cells to elicit appropriate physiological and biochemical responses (Dohlman *et al*., 1996). Typical units of the G protein‐coupled signalling system usually contain a G protein‐coupled receptor (GPCR), a G protein composed of α, β and γ subunits and various effectors (Li *et al*., 2007). The extensive cross‐talk between these G protein‐coupled signalling pathways forms a network that regulates a broad range of cellular processes, including transcription, motility, contractility and secretion, resulting in changes in systemic functions (Neves *et al*., 2002). In fungi, G proteins play critical roles in a variety of cellular functions related to vegetative growth and pathogenic development, including morphogenesis, conidiation, secondary metabolite production and virulence (Lengeler *et al*., 2000; Yu and Keller, 2005). Accordingly, RGSs primarily function as GTPase accelerators, which promote GTP hydrolysis by the Gα subunits, thereby inactivating the G protein and rapidly switching off G protein‐coupled signalling pathways (De Vries *et al*., 2000; Siderovski and Willard, 2005).

The availability of data on the whole‐genome of sequenced fungi made it possible to identify the encoding genes of RGS and RGS‐like proteins on a genomewide scale and to enrich our understanding of the properties and molecular mechanisms of the RGS and RGS‐like genes. There are generally four to eight RGS and RGS‐like genes in fungi, and their transcriptional direction is irregular (Wang *et al*., 2013). Generally, the RGS domain is the core of RGS proteins and each RGS protein typically contains one such domain (Wang *et al*., 2013). Interestingly, RGS domains can be found in conjunction with a variety of other domains, such as DEP for membrane targeting, phosphatidylinositol binding (Phox, PX) for phosphatidylinositol binding (IPR001683), Phox‐associated (PXA), which is associated with PX (IPR003114), and G protein gamma subunit‐like (GGL) for binding Gβ subunits (IPR001770) (De Vries *et al*., 2000). Among them, the DEP domain can mediate targeting of effectors and regulators to specific GPCRs and provides the means to dictate the nature, duration and specificity of the response (Ballon *et al*., 2006). Furthermore, the DEP domain might also be involved in targeting RGS proteins to the Golgi and plasma membranes (Burchett, 2000; Consonni *et al*., 2014), as well as the expression of a group of genes containing stress response elements in the promoter regions (Burchett *et al*., 2002). In this study, we first identified and confirmed eight RGS genes in the *V. dahliae* strain Vd8 with high sequence diversity, and, interestingly, structure–function analysis showed that VdRGS1 possessed tandem DEP domains, and its transcript level was significantly higher compared to other family members when induced with the roots of cotton seedlings, indicating that *VdRGS1* plays important roles in the virulence of *V. dahliae*.

### Functional analyses of RGS proteins in fungi

Recently, more and more RGS and RGS‐like proteins have been identified and characterized in a variety of fungi, and their analysis indicates that RGS proteins play pivotal roles in the upstream regulation of fundamental biological processes in fungi, including vegetative growth, mycotoxin/pigment production, sporulation, mating and pathogenicity (Park *et al*., 2012; Zhang *et al*., 2011). The yeast *S. cerevisiae* has four RGS and RGS‐like proteins: Sst2, Rgs2, Rax1 and Mdm1. Among them, Sst2 was identified through a screen for negative regulators of the pheromone response (Chan and Otte, 1982), and genetically and physically interacts with the Gα protein Gpa1, and can accelerate the GTPase activity of Gpa1 (Dohlman *et al*., 1996; Yi *et al*., 2003). In stationary phase cells, Rgs2 serves as a multicopy suppressor of Gα Gpa2‐dependent loss of heat shock resistance and has been shown to accelerate Gpa2 GTP binding and hydrolysis (Versele *et al*., 1999). Rax1 plays an important role in the establishment and maintenance of cell polarity (Fujita *et al*., 2004), and Mdm1 is required for the maintenance of proper nuclear and mitochondrial inheritance in cells growing at elevated temperatures (Fisk and Yaffe, 1997).

Moreover, the functions of RGS proteins have also been characterized in several plant pathogenic fungi, such as *M. oryzae*,* G. zeae* and *F. verticillioides*. *Magnaporthe oryzae* contains eight RGS and RGS‐like proteins, named MoRgs1 to MoRgs8. MoRgs1 and MoRgs4 positively regulate surface hydrophobicity, conidiation and mating. In addition, MoRgs4 also has a role in regulating laccase and peroxidase activities. MoRgs1, MoRgs2, MoRgs3, MoRgs4, MoRgs6 and MoRgs7 are important for germ tube growth and appressorium formation, and MoRgs1, MoRgs3, MoRgs4 and MoRgs7 are also required for full virulence (Zhang *et al*., 2011). *Fusarium verticillioides* has six RGS proteins (RgsA, RgsB, RgsC1, RgsC2, FlbA1 and FlbA2), and transcriptional analysis suggests that RgsB, FlbA1 or FlbA2 acts as a negative regulator of conidiation and fumonisin B1 (FB1, a toxin) biosynthesis, while, conversely, RgsC1 serves as a positive regulator (Mukherjee *et al*., 2011). Similarly, *G. zeae* contains seven RGS proteins (FgFlbA, FgFlbB, FgRgsA, FgRgsB, FgRgsB2, FgRgsC and FgGprK), and the phenotypes of deletion mutants suggest that they play versatile roles, including roles in conidia morphology, vegetative growth, mycotoxin production, sporulation, sexual development and virulence (Park *et al*., 2012). In this study, the phenotypes of deletion mutants further revealed that *VdRGS1* plays crucial roles in fungal conidiation, microsclerotium development, aerial hyphae production and virulence, which are key elements of VW. Based on this, *VdRGS1* plays important roles in the pathogenicity of *V. dahliae*, and there is potential to engineer this gene to improve cotton resistance to *V. dahliae*.

### Excavation of the virulence gene in *V. dahliae*

*Verticillium* wilt, caused by *V. dahliae*, is generally acknowledged as a destructive threat to a broad range of dicotyledonous host species. Many studies on *V. dahliae* have focused on its biological control and improved crop resistance (Conn *et al*., 2005; Debode *et al*., 2007; Fradin *et al*., 2011). However, relatively few studies have been carried out on genes involved in the pathogenicity of *V. dahliae*.

Elucidation of the signal transduction pathways that govern the development of microsclerotia and pathogenicity may be useful in designing novel control strategies for VW. For example, in the mitogen‐activated protein kinase (MAPK) signal cascade, *VMK1*, which encodes a MAPK, regulates virulence, conidiation and microsclerotia formation in *V. dahliae* (Rauyaree *et al*., 2005). In addition, VdHog1 affects the osmotic stress response, microsclerotia formation and virulence (Wang *et al*., 2016). *VdMsb* encodes a transmembrane mucin that is highly conserved in the MAPK signal pathway and plays a role in development and virulence in *V. dahliae* (Tian *et al*., 2014). Moreover, those homologous genes that are thought to play roles in G protein signalling, including cAMP‐dependent PKA pathway signalling (Tzima *et al*., 2010), and vesicle fusion (Yang *et al*., 2013). Among these components, VdPKAC1, the catalytic subunit of cAMP‐dependent PKA, regulates the microsclerotia production, indicating that the cAMP pathway antagonizes the VMK1 kinase‐mediated pathway in the regulation of microsclerotia formation (Tzima *et al*., 2010). The G protein β subunit controls virulence, microsclerotia formation, and conidiation in *V. dahliae* (Tzima *et al*., 2012). In addition, the transcription factor (TF) calcineurin‐responsive zinc finger (Crz1) serves as an important downstream regulator of Ca^2+^‐signalling transduction pathways and affects the cell membrane‐perturbing agent, sodium dodecyl sulphate, microsclerotia formation and the virulence of *V. dahliae* (Xiong *et al*., 2015). VdNoxB/VdPls1‐mediated ROS production activates VdCrz1 signalling through Ca^2+^ elevation in hyphopodia, infectious structures of *V. dahliae*, to control penetration peg formation during the initial colonization of cotton roots (Zhao *et al*., 2016).

Furthermore, many genes in *V. dahliae* regulate fungal development and pathogenicity (Luo *et al*., 2014). *VDH1* (Klimes and Dobinson, 2006; Klimes *et al*., 2008), *VdGARP1* (Gao *et al*., 2010) and *VdNLP* (Santhanam *et al*., 2013; Zhou *et al*., 2012) are required for vegetative growth, and *EG‐1* (Maruthachalam *et al*., 2011; Novo *et al*., 2006), *VdSSP1* (Liu *et al*., 2013), *Vta2* (Tran *et al*., 2014), VdSNF1 (Tzima *et al*., 2011), *VdMFS* (Kapoor *et al*., 2009; Maruthachalam *et al*., 2011) and *VdSge1* (Santhanam and Thomma, 2013) are necessary for successful penetration of host plants. *CPC1* acts a regulator of the cross‐pathway control of amino acid biosynthesis (Timpner *et al*., 2013), and *VdThi4* is involved in thiazole biosynthesis and DNA repair (Hoppenau *et al*., 2014), important functions in the process of adapting to the host's intracellular environment. In addition, TFs, through interaction with other TFs or molecular chaperones, play important roles in the virulence or pathogenicity of *V. dahliae* (Meshi and Iwabuchi, 1995; Pabo and Sauer, 1992). The fungal‐specific TF Zn (II)_2_ Cys_6_‐type *Vdpf* plays important roles in melanized microsclerotia formation, conidia production and pathogenicity (Luo *et al*., 2016). RGSs act as GTPase accelerators that promote GTP hydrolysis by the Gα subunits, thereby inactivating the G protein and rapidly switching off G protein‐coupled signalling pathways (Wang *et al*., 2013). In this study, *VdRGS1* was verified as a crucial gene in the control of virulence and microsclerotia formation. Excavation and functional analysis of virulence genes in *V. dahliae* could supply key HIGS targets for enhancing cotton VW resistance.

### HIGS of *VdRGS1* enhanced cotton VW resistance

Cotton VW is one of the most serious vascular diseases to affect this crop and results in severe reductions in cotton mass and yield worldwide (Bolek *et al*., 2005; Cai *et al*., 2009). To increase the limited amount of germplasm resources and rapidly improve resistance to fungal diseases, several new approaches have emerged in recent years. An increasing number of studies have experimentally validated the idea that HIGS is a powerful and promising approach for durable control of pathogenic fungi (Cheng *et al*., 2015; Koch *et al*., 2013; Panwar *et al*., 2013; Qi *et al*., 2017; Song and Thomma, 2016; Zhang *et al*., 2016). The RXLR effector Avr3a gene is largely responsible for virulence of oomycete plant pathogen *Phytophthora infestans*, and HIGS of Avr3a in the potato pathogen *P. infestans* imparts partial resistance to late blight disease (Sanju *et al*., 2015). Highly abundant message #34 (HAM34) and cellulose synthase (CES1) genes regulate the growth and sporulation of *Bremia lactucae*, and transgenic plants specifically expressing double‐stranded RNA targeting these genes inhibit the biotrophic pathogen causing downy mildew of lettuce (Govindarajulu *et al*., 2015). *PsFUZ7*, which encodes MAPKK, is an important pathogenicity factor that regulates infection and development of Pst, and HIGS of *PsFUZ7* confers stable resistance to wheat stripe rust (Zhu *et al*., 2017). Except for the effectiveness of HIGS approach, some reports also indicated that there was a randomness to silence the target gene due to the random 21–23nt production during the RNA interference process (Cheng *et al*., 2015; Song and Thomma, 2016) and need to further develop the stable transformants with high silencing efficiency of target gene.

In this study, we confirmed that *VdRGS1* was an important target gene for HIGS to enhance cotton VW resistance. Among eight RGS genes in *V. dahliae*,* VdRGS1* had the highest expression level and was also significantly induced when treating with cotton roots. Previous reports have shown that MoRgs1 in *M. oryzae* serves as a negative regulator of G protein signalling in the control of developmental processes such as surface hydrophobicity, conidiation, appressorium formation, mating and virulence (Liu *et al*., 2007; Zhang *et al*., 2011), and structure–function analysis of MoRgs1 found that it possessed specific vesicular/membrane targeting functions via N‐terminal DEP domains (Ramanujam *et al*., 2012). The structural features of VdRGS1 are similar to those of MoRgs1, and the phenotypes of our *VdRGS1* deletion and complementation mutants suggested that this gene played a variety of roles, including aerial hyphae development (Figure 3b), spore production (Figure S2b), microsclerotium formation (Figures 3c–e) and pathogenicity (Figure 4).

The virus‐mediated HIGS in planta‐generated RNAi serves as an effective strategy for functional analysis, as well as for controlling pathogen infection in a diverse range of plants (Panwar *et al*., 2013; Qi *et al*., 2017; Song and Thomma, 2016). BSMV‐mediated HIGS in the wheat leaf rust fungus *Puccinia triticina* (Pt) obviously suppressed the disease phenotype by targeting three predicted pathogenicity genes, a MAPK, a cyclophilin and a calcineurin regulatory subunit (Panwar *et al*., 2013). In addition, TRV‐based HIGS successfully silenced *Ave1* in *V. dahliae* and obviously inhibited VW disease in tomato (Song and Thomma, 2016). Here, we further tested the effectiveness of TRV‐mediated HIGS in cotton using VW fungus V991‐GFP by targeting the imported GFP genes, and the findings suggested that silencing of the GFP gene was successful, as shown by a reduction in the number of detectable GFP fluorescent spores of about 50% (Figures 5 and S1b). Further, we confirmed that TRV‐mediated HIGS targeting of *VdRGS1* in invading *V. dahliae* Vd8 and V991 strains significantly enhanced broad‐spectrum resistance to cotton VW (Figures 6, 7, S7 and S8). These findings suggest that TRV‐based HIGS in planta‐generated RNAi is an effective strategy for functional analysis, and TRV‐mediated HIGS of *VdRGS1* could effectively compromise *V. dahliae* infection in cotton plants. Based on TRV‐based HIGS results, we have constructed the different vectors for RNA interference of the *VdRGS1* against the invasive *V. dahliae*, to develop the stable transgenic cotton lines with high silencing efficiency of *VdRGS1* for improving the durable disease resistance in cotton. Hopefully, combining the strategy of overexpression of cotton disease‐resistant genes with HIGS of the key virulence genes in invading *V. dahliae* will be very efficient in the control of cotton VW.

## Experimental procedures

### Plant growth conditions and manipulations

Seedlings of *G. hirsutum* cv. Junmian 1 were grown in a controlled environment chamber under the conditions: 16‐h light/8‐h dark cycle at 28 °C for 2 weeks. Seedlings at the second true leaf stage were used for an infection assay, in which the growth conditions were changed to 25/23 °C (day/night), with a 16‐h light/8‐h dark cycle for disease symptoms observation. All necessary permits for collecting *G. hirsutum* cv. Junmian 1 were obtained from Nanjing Agricultural University, Jiangsu Province, China.

### Identification and structural analysis of RGS proteins in *V. dahliae*

Based the RGS (PF00615) domain, eight *VdRGS* genes were identified during a search of the *V. dahliae* strain VdLs17 genome database (http://genome.jgi.doe.gov/Verda1/Verda1.home.html) using HMMER (V3.0) software (Eddy, 2011) and Pfam (http://pfam.xfam.org/) (Finn *et al*., 2016) protein family databases, and these were further confirmed using the SMART (Letunic *et al*., 2012) and INTERPROSCAN (Hunter *et al*., 2012) programs. A phylogenetic tree was constructed by MEGA 5.2 (http://www.megasoftware.net/) using the neighbour‐joining method, and the bootstrap test was replicated 1000 times.

### *Verticillium dahliae* strains and culture conditions

The *V. dahliae* strain Vd8, which causes defoliation in cotton, was isolated from cotton seedlings in Jiangsu, China. This strain was used as a wild type and to generate the *VdRGS1* deletion mutants. All strains used in this study were stored long term at −80 °C as conidial suspensions in 15% glycerol. The *V. dahliae* strain V991 is a highly aggressive and defoliating strain and was introduced from Jiangsu Academy of Agricultural Sciences, Nanjing, China, and stored in our laboratory (State Key Laboratory of Crop Genetics & Germplasm Enhancement, Nanjing Agriculture University, Nanjing, China).

The *V. dahliae* strain Vd8 and its variants were initially grown on PDA medium (200 g potato, 20 g glucose, 15 g agar) at 25 °C in the dark. Following this initial growth, liquid CM containing 50 millilitre (mL) 20× nitrate salts, 1 mL 1000 × Trace, 1 mL vitamin solution, 10 g glucose, 2 g peptone, 1 g yeast extract, 1 g casamino acids was used to collect vegetative hyphae. Antibiotic‐resistant strains were grown on PDA or CM medium amended with hygromycin (25 μg/mL) or geneticin (50 μg/mL) as appropriate.

### Gene expression and phenotypic analysis in *V. dahliae*

For detecting the expression of *V. dahliae* genes when induced by the root of cotton seedlings, 18‐bottle spore suspensions of *V. dahliae* strain Vd8 with the same concentration (1 × 10^7^ conidia/mL) for six induced time points, each three biological repeats, were prepared. The roots of the three cotton seedlings for every bottle were dipped into the *V. dahliae* spore suspensions, respectively. Then, the roots were taken out and the *V. dahliae* spores were washed down from the cotton roots surface into the original spore suspensions using sterilized water at different induced time points, including 0, 2, 4, 6, 12 and 24 h. The collected spore samples with three biological repeats were used to extract mRNA for detecting the expression patterns of RGS genes.

Differences in colony morphology and hyphal growth in the *V. dahliae* strain Vd8 and its variants were also assessed by incubation on PDA or CM medium at 25 °C. For investigating the spore germination, spore suspensions of the Δ*VdRGS1*,* VdRGS1*‐com and wild‐type strain Vd8 with the same growth period and concentration were prepared and observed by confocal microscopy (LSM710; Zeiss, Jena, Germany), and then, 1000 spores of the different strains were used to calculate the conidial germination with three repeats. For microscopic observation of hyphal growth on solid medium, the single spores of Δ*VdRGS1*,* VdRGS1*‐com and wild‐type strain Vd8 were cultured for 24 and 36 h on plates, respectively, and observed under microscope (DM2500; Leica, Wetzlar, Germany). For conidial production tests, conidia were harvested from CM, and then, 1000 spores of the different strains were respectively incubated in liquid Czapek–Dox medium (CPK) with three repeats on a shaking table at 150 rpm per minute at 25 °C for 7 days. Single spores from each strain were grown on CM medium at 24 °C for 5 days to observe microsclerotia production. In addition, the conidia were harvested from the CM medium and adjusted to a concentration of 1 × 10^4^ spores/mL, and then, 10 μL of the conidial suspension was sprayed onto a cellulose membrane (Ø = 80 mm; pore size = 0.22 μm) that had been overlaid on solid BM containing 10 g/L glucose, 0.2 g/L sodium nitrate, 0.52 g/L KCl, 0.52 g/L MgSO_4_·7H_2_O, 1.52 g/L KH_2_PO_4_, 3 μm thiamine HCl, 0.1 μm biotin, 15 g/L agar. Distinct microsclerotia formations of the different strains at 5 days postincubation were observed under a light microscope (DM2500; Leica, Wetzlar, Germany).

### Nucleic acid manipulations and DNA blots

Genomic DNA of the *V. dahliae* strains was extracted using the cetyltrimethyl ammonium bromide method, which was described previously by O'Donnell *et al*. (1997). Fungal strains were grown in liquid CPK medium (Wang *et al*., 2004b) for 5–7 days with shaking at 150 rpm at 25 °C. Subsequently, the mycelia were harvested and lyophilized for DNA extraction. The DIG High Prime DNA Labeling and Detection Starter Kit I was used for the Southern blot analysis according to the manufacturer's protocol (Roche, Basel, Switzerland). Genomic DNA of wild‐type fungi, and the *VdRGS1* deletion and complementation mutants, was digested with the restriction enzyme SacI for DNA blot analysis and about 5 μg of each was loaded on a 0.8% agarose gel. The probe location was selected upstream of *VdRGS1*, and amplified and sequenced from genomic DNA with primers. All primers used in this study are shown in Table S1.

Total RNA was isolated from mycelia using TRIzol Reagent (Invitrogen, Carlsbad, USA) according to the manufacturer's instructions. RNA samples (2 μg per reaction) were reversely transcribed into cDNA with the HiScript Q RT SuperMix for qPCR (+gDNA wiper) (Vazyme, Nanjing, China).

### Cloning and qPCR analysis of the RGS genes in *V. dahliae*

Based on the known sequences of *V. dahliae* strain VdLs17 available from the genome database (http://genome.jgi.doe.gov/Verda1/Verda1.home.html), gene‐specific primers (Table S1) were designed using Primer 5.0 software (PREMIER Biosoft, Palo Alto, USA) to amplify the homologous genes of RGS with complete open‐reading frames in the Vd8 strains. High‐fidelity ExTaq DNA polymerase (Takara Biotechnology [Dalian] Co. Ltd., Dalian, China) was used in standard PCRs, and all the PCR products were cloned into pMD19‐T cloning vectors (Takara, Dalian, China) and transformed into *E.coli* DH5α bacterial strains obtained from our laboratory (State Key Laboratory of Crop Genetics & Germplasm Enhancement, Nanjing Agriculture University, Nanjing, China). At least five clones per gene were randomly selected and sequenced.

For the quantitative real‐time PCR (qPCR) analysis, gene‐specific primers (Table S1) were designed using Beacon Designer 7.0 (PREMIER Biosoft, Palo Alto, USA), and β‐tublin (KF555285.1) was used as a reference gene. Real‐time PCR amplification reactions were performed on an ABI 7500 Real Time PCR System (Applied Biosystems, Carlsbad, USA) using AceQ SYBR Green Master (Low Rox Premixed) (Vazyme) with three technical replicates for each biological sample. Expression data from three biologically independent experiments were analysed and presented as means ± SD.

### *VdRGS1* deletion and complementation

The flanking DNA sequences of the entire ORF were used to design PCR primers to clone the full‐length sequences of *VdRGS1* from *V. dahliae* strain Vd8. The amplified products were cloned into pMD19‐T vector (Takara Biotechnology [Dalian] Co. Ltd., Dalian, China) for sequencing. The *Agrobacterium tumefaciens*‐mediated transformation method was used to generate gene deletion mutants by targeted gene replacement. To construct the gene replacement vector, pDH‐*VdRGS1*, about 1–1.5 kb of the upstream (5′) and downstream (3′) flanking sequences of *VdRGS1* were amplified. Next, both *VdRGS1* flank amplicons were inserted up‐ and downstream of the hygromycin‐resistance cassette (HPH) of the pDHt2 vector (Figure 2a). The gene complementary vector, pCOM‐*VdRGS1*, was constructed by inserting a 4.1‐kb fragment containing the *VdRGS1* coding sequence and the native promoter and terminator sequences (Zhou *et al*., 2013). A GFP expression vector was also constructed to obtain Vd8‐ and V991‐GFP transformants. All constructs were introduced into *Agrobacterium* strain AGL1 for fungal transformation. Next, the transformants were screened on PDA medium supplemented with antibiotics, either 25 μg/mL of hygromycin B (for gene replacement) or 25 μg/mL of G418 geneticin (for gene complementation), basing on the selection marker in the respective plasmid vectors. The presence of the target gene and HPH gene was verified by PCR or RT‐PCR using the corresponding specific primers. The β‐tublin (KF555285.1) gene was used as an internal control, and *VdRGS1* deletion mutants were also validated by DNA sequencing of the gene locus (Figure 2c).

### Pathogenicity assays

Virulence assays of V991 and Vd8 strains, and *VdRGS1* deletion and complementation mutants, were evaluated using *G. hirsutum* cv. Junmian 1 (susceptible) seedlings. The different strains were incubated on CM or PDA medium at 25 °C for 4–7 days and inoculated into 100 mL CPK liquid medium with shaking for about 7 days. The spore concentration was adjusted to about 1 × 10^7^ conidia/mL with sterile water. For the pathogenicity assay with cotton, more than 40 plants (per isolate) were inoculated by immersing their roots in 20 mL conidial suspension, and the infection assays were performed three times.

Disease symptoms were recorded starting at 10 days postinoculation with Vd8 or V991 strains, until all the leaves of the plants were defoliated. Statistical analyses were used to compare the average percentage of diseased leaves in experimental plants with that in controls. According to the average percentage of diseased leaves, the disease grade was classified as follows: 0 (no symptoms), 1 (0%–25% wilted leaves), 2 (25%–50%), 3 (50%–75%) and 4 (75%–100%) (Liu *et al*., 2014). For cotton plants, the DI was calculated according to the following formula: DI = [(∑disease grades × number of infected plants)/(total checked plants × 4)] × 100% (Wang *et al*., 2004a). The data from three biologically independent experiments were analysed and presented as means ± SD. In addition, statistical significance was determined by Student's *t*‐tests.

To analyse invasive growth in cotton, the stems of plants infected with *V. dahliae* strains or mutants were cut and observed using a stereoscope (Olympus MVX10, Tokyo, Japan).

For biomass quantification in planta, stems of three inoculated plants were harvested at 15 dpi. The samples were ground to powder and genomic DNA was isolated (Santhanam *et al*., 2013). The fungus‐specific primer ITS‐F, which is based on the internal transcribed spacer region of the ribosomal DNA, in combination with the *V. dahliae*‐specific reverse primer ST‐Ve1‐R was used to measure fungal colonization. Primers for cotton histone 3 (AF024716) were used as endogenous plant controls. Real‐time PCR was carried out on genomic DNA using an ABI7500 PCR machine (Applied Biosystems) in combination with SYBR Green Master (Low Rox Premixed) (Vazyme), with three technical replicates for each biological sample, as previously described (Santhanam *et al*., 2013).

### TRV treatment

The pTRV1 and pTRV vectors used for VIGS analysis were generously provided by Dr. Libo Shan of Texas A & M University (College Station, TX). We constructed TRV: *VdRGS1‐1*, TRV: *VdRGS1‐2*, TRV: *VdRGS1‐3*, TRV: *VdRGS1‐4*, TRV: *GFP*, and TRV: *GhCLA1* vectors. These vectors were transformed into *A. tumefaciens* strain GV3101, which was obtained from our laboratory (State Key Laboratory of Crop Genetics & Germplasm Enhancement, Nanjing Agriculture University, Nanjing, China). Subsequently, all the TRV vectors were agroinfiltrated as previously described (Gao *et al*., 2011a,b, 2013; Xu *et al*., 2017). Briefly, cotyledons of 8‐day‐old Junmian 1 cotton seedlings were infiltrated with 1:1 mixtures of pTRV1 and pTRV constructs. All plants were grown in the same growth chamber at 23/21 °C (day/night), with a 16‐h light/8‐h dark cycle, and changes in plant phenotypes were observed.

About 2 weeks after TRV: *GhCLA1* inoculation, the plants showed highly uniform bleaching in newly emerged leaves (Figure S4). Next, the control and VIGS plants were dip‐inoculated with Vd8 or V991 conidia suspension (1 × 10^7^ conidia/mL), as described previously (Wang *et al*., 2011). About 2 weeks after inoculation, the control plants displayed obvious leaf‐yellowing phenotypes. Next, we randomly and repeatedly cut and incubated the stems on PDA for 3 days for RNA extraction.

To further confirm the feasibility of TRV‐mediated HIGS in cotton plants by targeting the predicted pathogenicity genes of *V. dahliae*, the control, TRV: 00 and TRV: *GFP* plants were dip‐inoculated with V991‐GFP conidia suspension and the detectable GFP fluorescence of spores was observed. Briefly, the stems of the control, TRV: 00 and TRV: *GFP* plants dip‐inoculated with V991‐GFP conidia were cut and grown on CM or PDA medium 25 °C for 2–3 days, and GFP fluorescence in the conidia suspension was observed by confocal microscopy (LSM710; Zeiss, Jena, Germany). 1000 spores each for the different treatments were individually used to calculate the *GFP*‐silenced efficiency with three repeats.

## Competing interests

The authors declared that they had no competing interests.

## Authors’ contributions

WG conceived the original screening and research plans; JX and WG performed most of the experiments; XW, YL, JZ, GW and CD provided technical assistance; WG, JX and XW designed the experiments and analysed the data; WG, JX and XW conceived the project and wrote the article with contributions of all the authors; and WG and JX supervised and complemented the writing.

## Supporting information


**Figure S1** Average number of spores in the different treatments.

**Figure S2** Effects of *VdRGS1* deletion on colony morphology and spore production.

**Figure S3** Pathogenicity assays of the V991 and V991‐GFP strains on Junmian 1 seedlings.

**Figure S4** Silencing of the endogenous cloroplastos alterados gene (*GhCLA1*) in cotton through VIGS.

**Figure S5** Expression of the *GFP* gene in the invading V991‐GFP stain among the control, TRV: 00 and TRV: *GFP* plants.

**Figure S6** Alignment of *VdRGS1* cDNA sequences from Vd8 and V991 strains respectively.

**Figure S7** Assessment of disease index (DI) in the VIGS plants inoculated Vd8 and V991 at 20 dpi.

**Figure S8** Fungal biomass detection in cotton plants at 15 dpi.

**Table S1** Information on PCR primers used in this study.
