# Supplementary figures and images for: Host‐induced gene silencing of a regulator of G protein signalling gene (VdRGS1) confers resistance to Verticillium wilt in cotton
Source: Plant Biotechnol J. 2018 Mar 5;16(9):1629–43. doi: 10.1111/pbi.12900 (PMC6096726; doi:10.1111/pbi.12900)

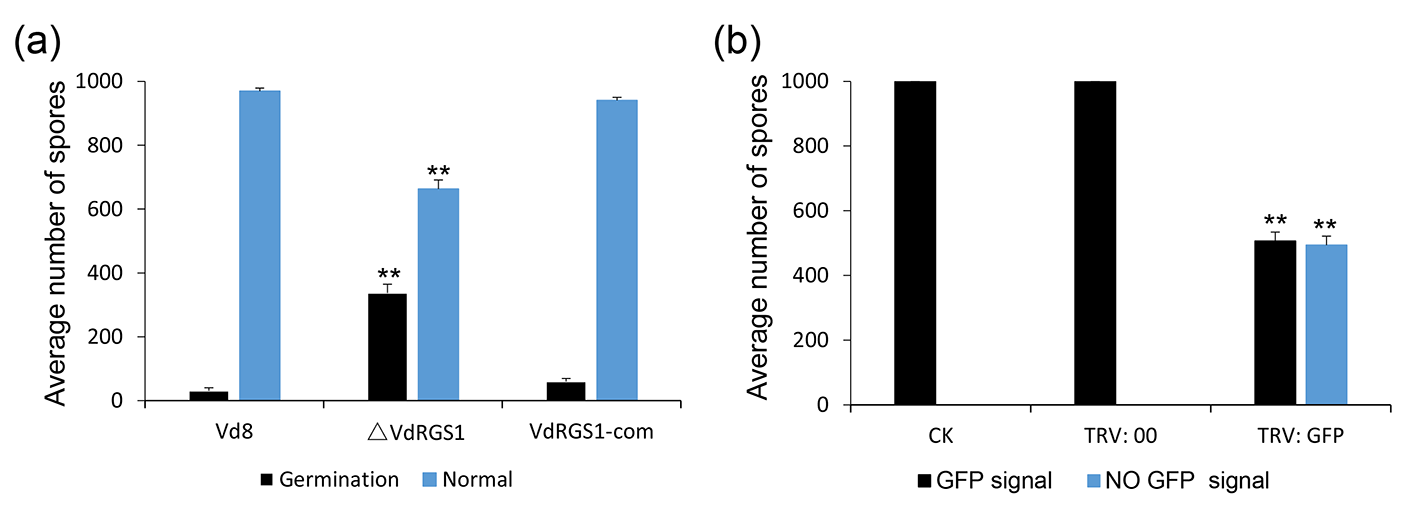

Supplement: Supplementary file 1 — Figure S1 Average number of spores in the different treatments. [file PBI-16-1629-s004.tif]

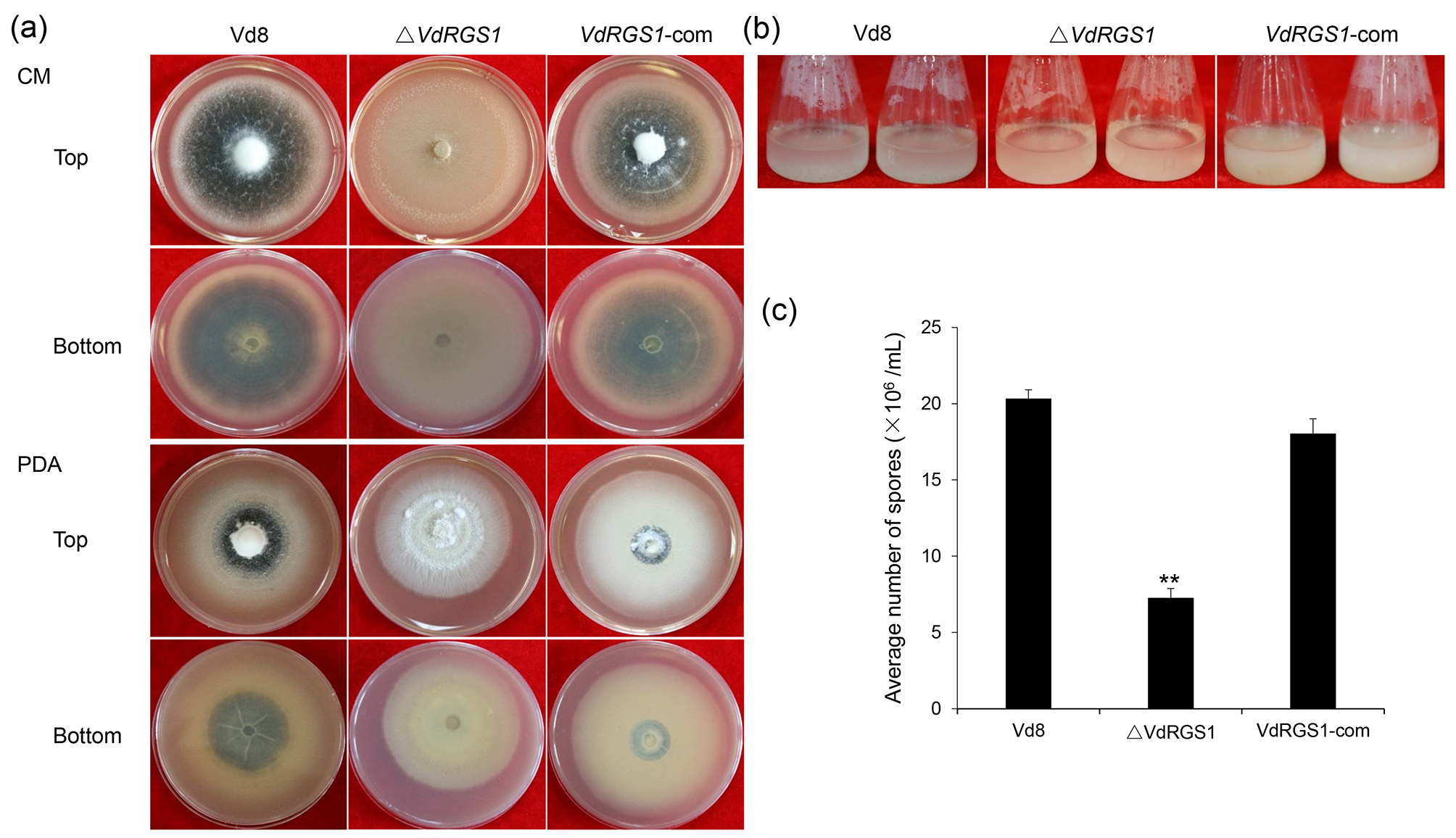

Supplement: Supplementary file 2 — Figure S2 Effects of VdRGS1 deletion on colony morphology and spore production. [file PBI-16-1629-s007.tif]

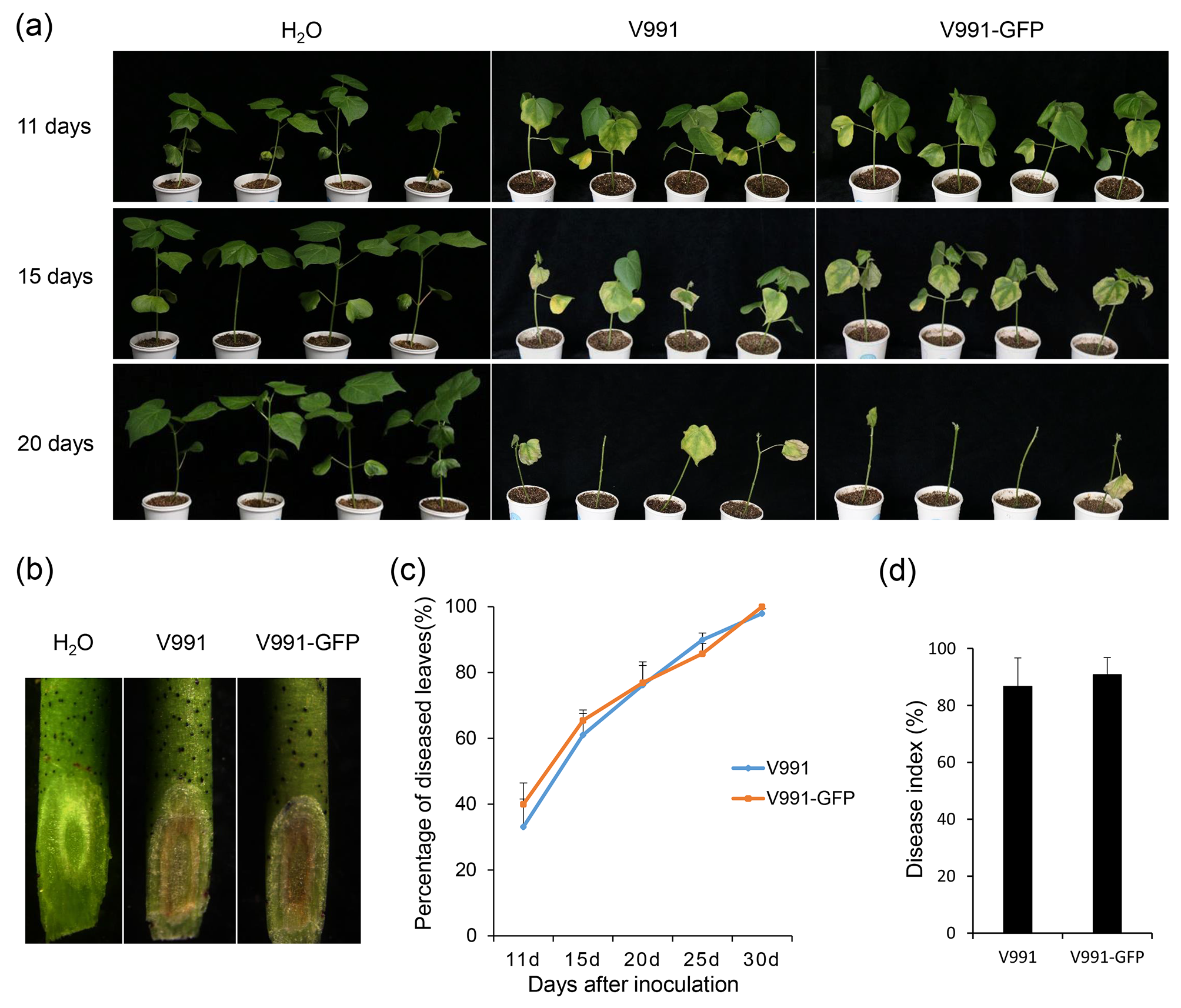

Supplement: Supplementary file 3 — Figure S3 Pathogenicity assays of the V991 and V991‐GFP strains on Junmian 1 seedlings. [file PBI-16-1629-s006.tif]

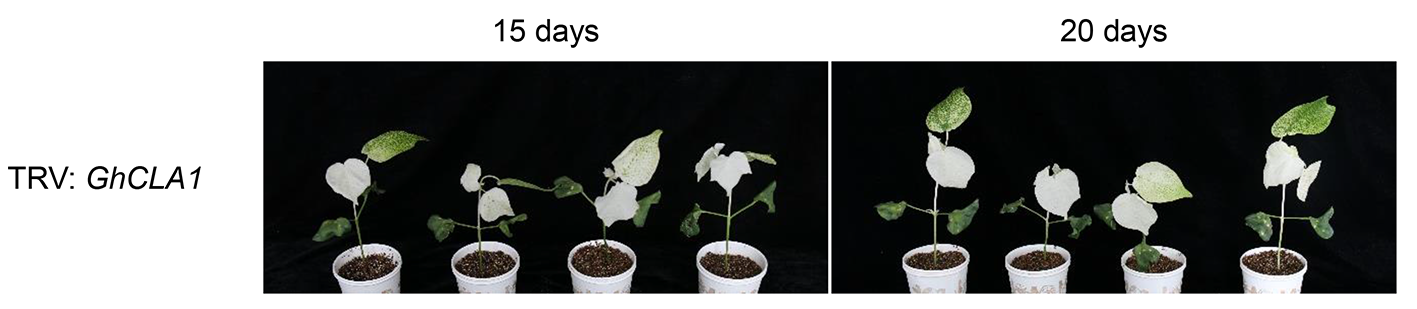

Supplement: Supplementary file 4 — Figure S4 Silencing of the endogenous cloroplastos alterados gene (GhCLA1) in cotton through VIGS. [file PBI-16-1629-s008.tif]

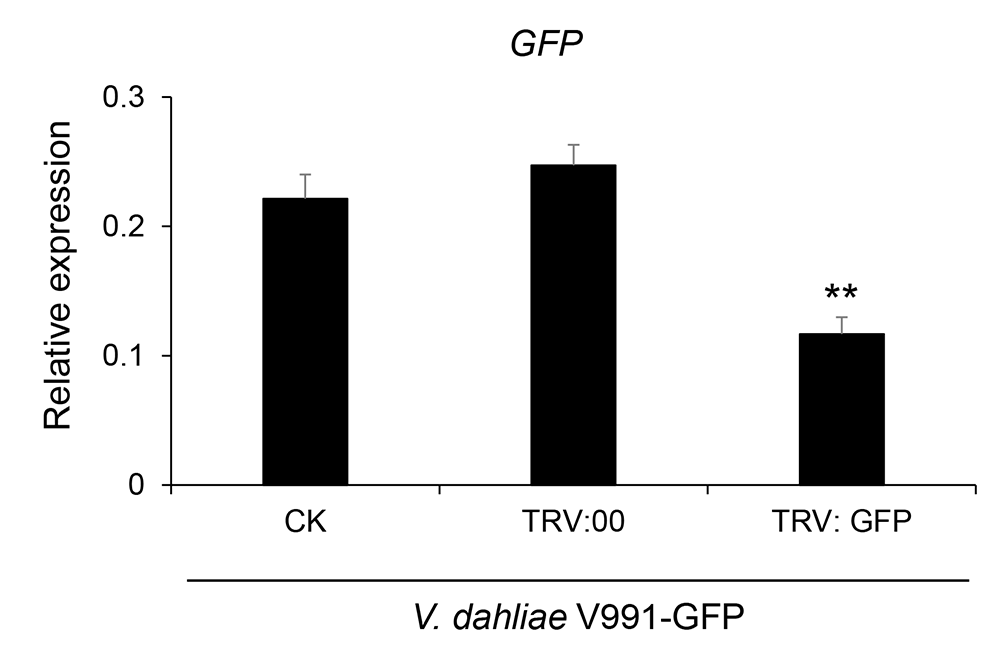

Supplement: Supplementary file 5 — Figure S5 Expression of the GFP gene in the invading V991‐GFP stain among the control, TRV: 00 and TRV: GFP plants. [file PBI-16-1629-s001.tif]

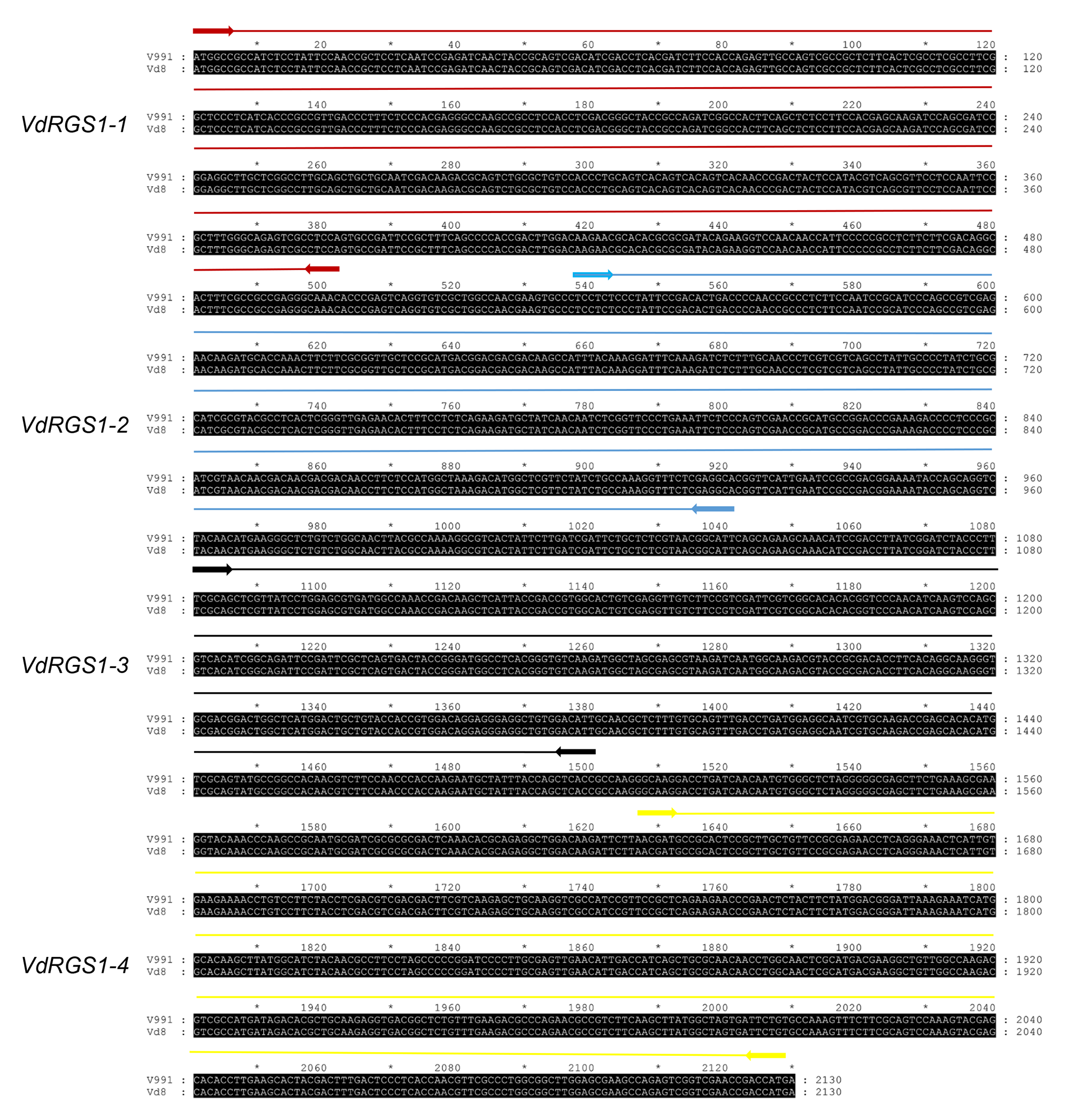

Supplement: Supplementary file 6 — Figure S6 Alignment of VdRGS1 cDNA sequences from Vd8 and V991 strains respectively. [file PBI-16-1629-s002.tif]

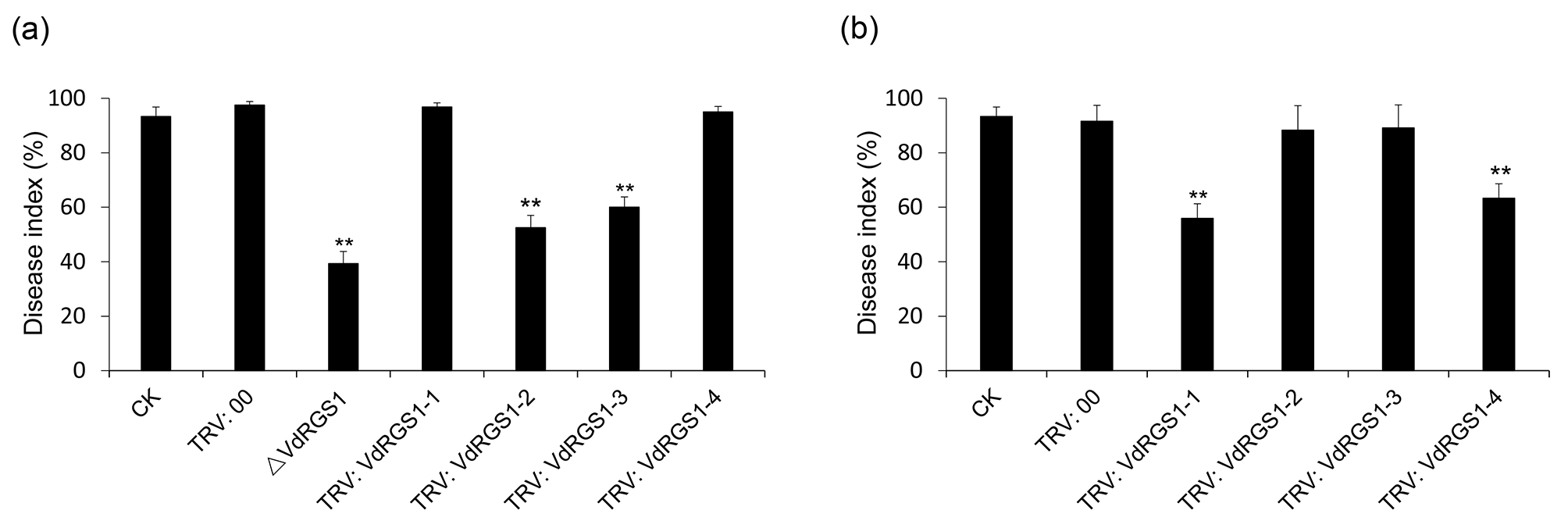

Supplement: Supplementary file 7 — Figure S7 Assessment of disease index (DI) in the VIGS plants inoculated Vd8 and V991 at 20 dpi. [file PBI-16-1629-s003.tif]

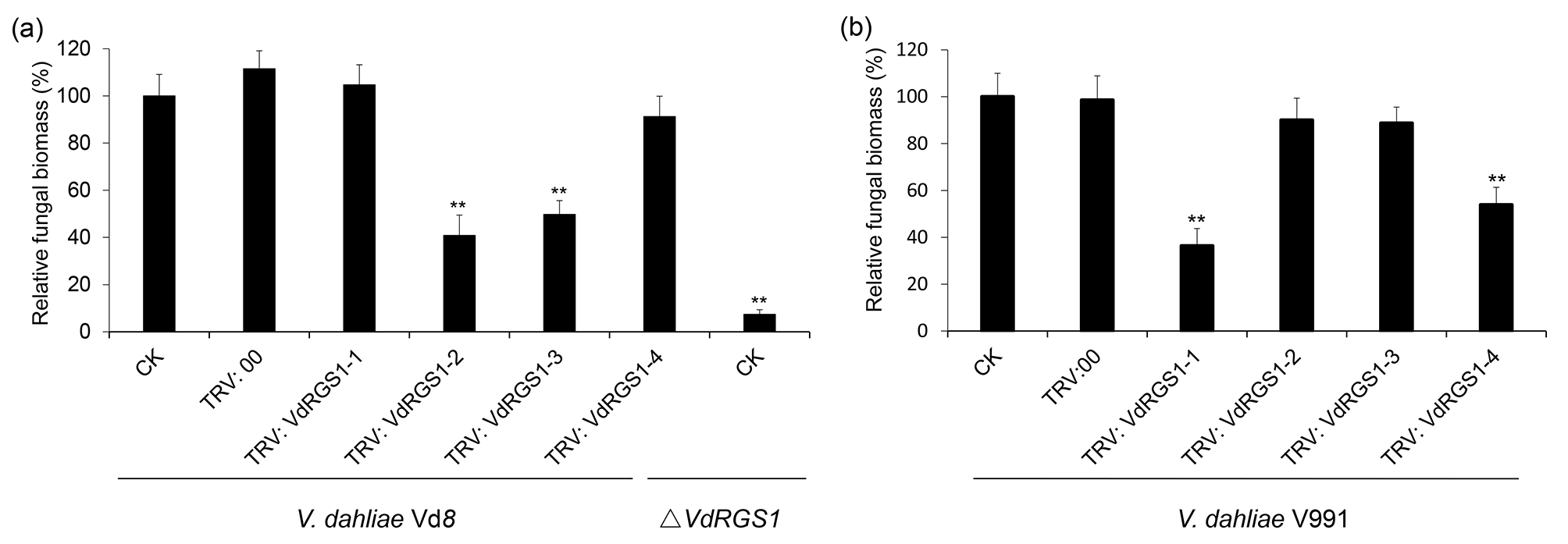

Supplement: Supplementary file 8 — Figure S8 Fungal biomass detection in cotton plants at 15 dpi. [file PBI-16-1629-s009.tif]
