# Supplementary material for: Host‐induced gene silencing of a regulator of G protein signalling gene (VdRGS1) confers resistance to Verticillium wilt in cotton
Source: Plant Biotechnol J. 2018 Mar 5;16(9):1629–43. doi: 10.1111/pbi.12900 (PMC6096726; doi:10.1111/pbi.12900)
Supplement: Supplementary file 9 — Table S1 Information on PCR primers used in this study. [file PBI-16-1629-s005.docx]

| **Table S1 Information on PCR primers used in this study** | | |  |
| --- | --- | --- | --- |
| Primer name | Sequence information（5′-3′） | Purpose | |
| S1039F | ATGGCCGCCATCTCCTATTC | *VdRGS1* cloning | |
| S1039R | TCATGGTCGGTTCGACCGAC |  | |
| S1040F | ATGGCTCTCTCAAAAAGAGAC | *VdRGS2* cloning | |
| S1040R | TCAGAGTGAAGGCAAGATGAG |  | |
| S1041F | ATGGAAGACACACATCCCGAC | *VdRGS3* cloning | |
| S1041R | CTACAATCTCTTGCCTGGAAC |  | |
| S1042F | ATGGAGGTCCCAACGTGGCT | *VdRGS4* cloning | |
| S1042R | TTACAGCTTGACAGTCTCCT |  | |
| S1043F | ATGGATCCCATGGCTGGCCTC | *VdRGS5* cloning | |
| S1043R | TCAGAGGAGTCGCTTGAAAAAG |  | |
| S1044F | ATGGGTCTCCTTTCGCTGAC | *VdRGS6* cloning | |
| S1044R | TTACATCAGTTTCCCCCCCG |  | |
| S1045F | ATGGCGCCCAAACACATGGT | *VdRGS7* cloning | |
| S1045R | TTACTCCTTTGCGAACGCCT |  | |
| S1046F | ATGGATTACTTGGTCTATGTC | *VdRGS8* cloning | |
| S1046R | TCACATCACCTTCATCGTCCA |  | |
| S1200F | CTCACAGTCACAGTCACA | *VdRGS1* for qRT-PCR | |
| S1200R | ATGGTTGTTGGACCTTCT |  | |
| S1203F | GCTGCTCTGGAAGTCTAT | *VdRGS2* for qRT-PCR | |
| S1203R | GTCCGTTACCGAGTCATAT |  | |
| S1205F | TACTTCCTCGCATCATACC | *VdRGS3* for qRT-PCR | |
| S1205R | GAATAGTGACGGCAAGAAC |  | |
| S1206F | TCATCTGGACTTGGATTGT | *VdRGS4* for qRT-PCR | |
| S1206R | GTTGCGTGAAGACTTGAG |  | |
| S1209F | AACATCTACACGCACAGTA | *VdRGS5* for qRT-PCR | |
| S1209R | TAAAGGCAGCAGGAATCT |  | |
| S1210F | CAACGAGAAGAGCAACAG | *VdRGS6* for qRT-PCR | |
| S1210R | TCATAATCCTTGATCCAGAGAA |  | |
| S1213F | CGAGACGAGCATCAAGTA | *VdRGS7* for qRT-PCR | |
| S1213R | GCACCACCATATCAATCAG |  | |
| S1215F | GTCAGAGTTCTCCAGGAC | *VdRGS8* for qRT-PCR | |
| S1215R | TCACATCACCTTCATCGT |  | |
| S4832F | CGCACCATCTTCTTCAAG | *GFP* for qRT-PCR | |
| S4832R | TGTGGCTGTTGTAGTTGTA |  | |
| W1596F | TTCCTTCACTGGTACACT | VdTubulin acting as reference gene for qRT-PCR | |
| W1596R | CTCCTCCTCCTCCTCATC |  | |
| W1598F | CCCTCAAGCTGTCCAACCC | VdTubulin acting as reference gene for RT-PCR | |
| W1598R | ATCTCGTCCATACCCTCACCA |  | |
| W9869F | CCGCTCGAGGTGAAATGCTCTTCGGTGGT | For amplifying the 5′flanking regions of *VdRGS1* | |
| W9869R | CCGGAATTCCGAAGGTTGAGGGTGGGT |  | |
| W9872F | CCGGAATTCCATACTCCCCGCATTTACCC | For amplifying the 3′flanking regions of *VdRGS1* | |
| W9872R | CCCAAGCTTGAGGATGGCTCAACATTTATGG |  | |
| S4344F | ATTATTATGGAGAAACTCGAGAAGCGCCCACTGAGAAGACTG | For generation of *VdRGS1* complementation plasmid | |
| S4344R | ACGACGGCCAGTGCCAAGCTTTCCCTTCCTATTCCCTGCCG |  | |
| S291F | TCTTGCGAATCCCAAACCTAC | For the DNA sequencing of the *VdRGS1* locus | |
| S291R | ACTGATTGCGAGATGAGGATGA |  | |
| S2886F | CGAGCGTAAGATCAATGGC | *VdRGS1* gene specific primers | |
| S2886R | TGAGGGAGTCAAAGTCGTAGTG |  | |
| Y7625F | GCCCTTCCTCCCTTTATTTC | For checking internal to HPH in transformants | |
| Y7625R | GATGTTGGCGACCTCGTATT |  | |
| S4426F | TCTCAGTTTGGTTGGCTTGC | For cloning the probe of Southern blot analysis | |
| S4426R | AGGGTGTTCGTTCCTTGGCT |  | |
| S4128F | CGAGACCGATAAGCGTTA | VDAG_03674 for qRT-PCR | |
| S4128R | GTGTGAAGTTGATGAAGATGT |  | |
| S4129F | GCCTATTATCTCACCTCTTCT | VDAG_00190 for qRT-PCR | |
| S4129R | GTTGATTGCTCCTGATTGTT |  | |
| S4130F | TGGCATCAAGACAGACATGTA | VDAG_03665 for qRT-PCR | |
| S4130R | ACAACGCGAGCGATGTCGAT |  | |
| S4131F | AAAGGTGTTTGAGAGCGGAC | VDAG_03393 for qRT-PCR | |
| S4131R | ATCTCCCTCTCCACAACAGC |  | |
| S4132F | TATGTCCCTGGCGGCTTTAA | VDAG_00183 for qRT-PCR | |
| S4132R | TGATCCACTCGCAGTCTTCA |  | |
| S4067R | GTGAGTAAGGTTACCGAATTCATGGCCGCCATCTCCTATTC | For constructing TRV: *VdRGS1-1* vector | |
| S4067R | CGTGAGCTCGGTACCGGATCCTTGCCCTCGGCGGCGAAA |  | |
| S4068F | GTGAGTAAGGTTACCGAATTCACACCCGAGTCAGGTGTCGC | For constructing TRV: *VdRGS1-2* vector | |
| S4068R | CGTGAGCTCGGTACCGGATCCCTTTTGGCGTAAGTTGCCAGA |  | |
| S4069F | GTGAGTAAGGTTACCGAATTCGCGTCACTATTCTTGATCGATTCTG | For constructing TRV: *VdRGS1-3* vector | |
| S4069R | CGTGAGCTCGGTACCGGATCCGGTGAGCTGGTAAATAGCATTCTTG |  | |
| S4070F | GTGAGTAAGGTTACCGAATTCACGATGCCGCACTCCGCT | For constructing TRV: *VdRGS1-4* vector | |
| S4070R | CGTGAGCTCGGTACCGGATCCTGGTCGGTTCGACCGACTC |  | |
| S4650F | GTGAGTAAGGTTACCGAATTCAGCACGACTTCTTCAAGTCCGC | For constructing TRV: *GFP* vector | |
| S4650R | CGTGAGCTCGGTACCGGATCCACGACGGCCAGTGCCAAG |  | |
| Y8991F | CGGTGGTGTGAAGAAGCCTCAT | Cotton histone3 (fungal biomass) | |
| Y8991R | AATTTCACGAACAAGCCTCTGGAA |  | |
| ITS1-F | AAAGTTTTAATGGTTCGCTAAGA | *V. dahliae* (fungal biomass) | |
| STVe1-R | CTTGGTCATTTAGAGGAAGTAA |  | |
